# Toward the Scale-Up of a Bicyclic Homopiperazine via Schmidt Rearrangement and Photochemical Oxaziridine Rearrangement in Continuous-Flow

**DOI:** 10.1021/acs.oprd.0c00361

**Published:** 2020-10-15

**Authors:** Michael Brown, Mohammed Aljarah, Hannah Asiki, Leo M. H. Leung, Deborah A. Smithen, Natalie Miller, Gabor Nemeth, Lawrence Davies, Dan Niculescu-Duvaz, Alfonso Zambon, Caroline Springer

**Affiliations:** † Drug Discovery Unit, Cancer Research UK Manchester Institute, University of Manchester, Alderley Park, Macclesfield SK10 4TG, United Kingdom; ‡ Cancer Research UK Centre for Cancer Therapeutics, The Institute of Cancer Research, 15 Cotswold Road, London SM2 5NG, United Kingdom

**Keywords:** homopiperazine, continuous-flow, Schmidt, oxaziridine, photochemical, microreactor

## Abstract

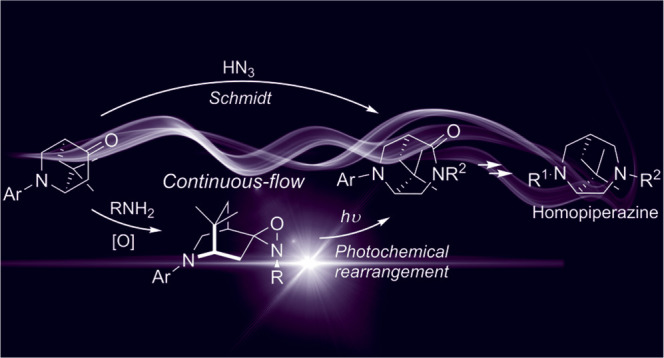

The scale-up of a chiral bicyclic homopiperazine of pharmaceutical interest was investigated. The outcome and safety profile of a key batch ring-expansion step via Schmidt rearrangement was improved using continuous-flow chemistry. The selectivity of nitrogen insertion for the ring expansion was improved via an alternative photochemical oxaziridine rearrangement under mild conditions, which when converted to continuous-flow in a simple and efficient flow reactor allowed the first photochemical scale-up of a homopiperazine.

## Introduction

**Figure 1:**
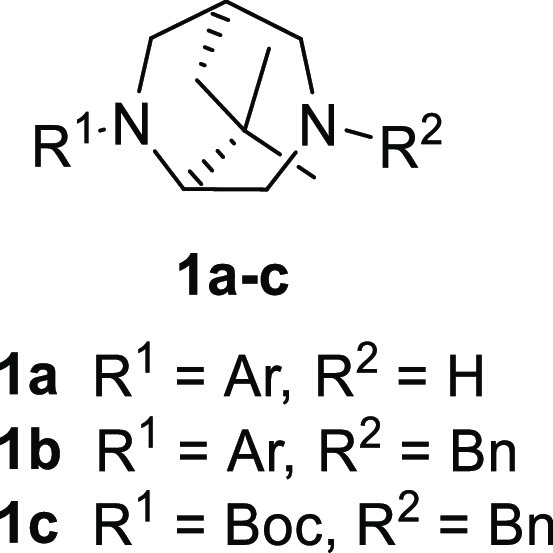
3,6-Diazabicyclo[3.2.2]nonanes (bridged homopiperazines).

Seven-membered heterocycles are important structures in pharmaceutical and industrial chemistry.^1^ Those with two nitrogen atoms in a 1,4-relationship are of particular pharmaceutical interest and include the top-selling benzodiazepine class of central nervous system active drugs. As part of our cancer research program into lysyl oxidase (LOX) inhibitors, we required a scalable route to chiral 3,6-diazabicyclo[3.2.2]nonanes (bridged homopiperazines) **1** (Figure 1).^2^ The configuration of the dimethyl-substituted bridge of this stereoisomer imparted superior stability to our inhibitors against in vivo metabolism compared with a range of bridged homopiperazine isomers. This conformationally restricted heterocycle, which could be accessed in either enantiomeric form, represents a versatile building block of general interest for drug discovery.

**Scheme 1:**
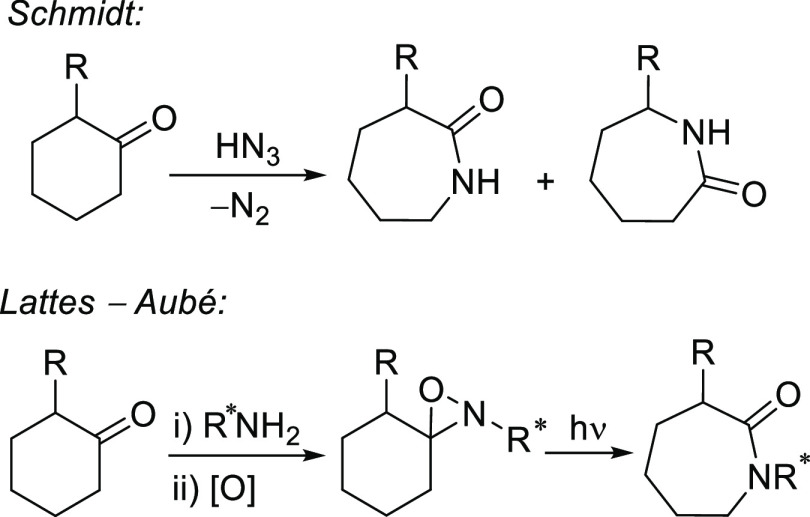
Ring Expansion via Schmidt and Oxaziridine Rearrangements

Despite the important biological activities of fused diazepines, the homopiperazine ring system is somewhat under-represented in drugs,^3^ in part because of the absence of general or scalable methods for its synthesis. Ring expansion by nitrogen insertion of more readily accessible six-membered rings is a commonly used strategy.^4,5^ The manufacture of ε-caprolactam, the key precursor for Nylon-6, involves a ring expansion via Beckmann rearrangement of cyclohexanone oxime and highlights an important industrial application of this approach.^6^ The related Schmidt rearrangement converts cyclic ketones to ring-expanded lactams using hydrazoic acid (HN_3_) generated in situ from sodium azide and a strong acid (Scheme 1).^7−9^ It is atom-economical and performs this transformation in a single step without the isolation of the intermediate oxime that is usually required in the Beckmann rearrangement. Hydrazoic acid is a toxic, volatile gas with explosive properties, and as such, its generation on scale in batch reactors must be carefully controlled.

Recent advances in flow chemistry, in particular microreactor technology, have demonstrated the safe utilization of hazardous reagents such as HN_3_ within small-volume enclosed reactors such that only small quantities are present at any one time.^10−12^ Recently reported was a Schmidt protocol using methanesulfonic acid–1,2-dimethoxyethane (MsOH–DME)^13^ that proved to be efficient at rearrangements of cyclohexanone and a range of acetophenones.^14^ Rearrangements of aryl carboxylic acids using superacidic TMSN_3_/triflic acid conditions,^15^ microwave-assisted intramolecular Schmidt rearrangements,^16^ and Beckmann rearrangements using trifluoroacetic acid^17^ and visible light^18^ have also recently been demonstrated in continuous-flow microreactors, providing safer protocols and precedent for the scalable use of these chemistries.

For unsymmetrical ketones (Scheme 1), nitrogen insertion reactions can lead to a mixture of lactam products that is dependent on the migration propensity of the carbonyl substituents. Additionally, bridged bicyclic ketones can give rise to mixtures of lactams from competitive methylene and bridgehead methine migration.^19^ The reaction outcomes are heavily influenced by the substrate: whereas Beckmann rearrangements of the oximes derived from bicyclo[2.2.2]octanone and the related 2-azabicyclo[2.2.2]octanones give exclusively bridgehead migration products,^20,21^ Schmidt reactions of bicyclo[2.2.2]octanone give solely the methylene migration product,^22^ and Schmidt reactions of 2-azabicyclo[2.2.2]octanones give rise to mixtures.^23^ There is a single report of the Schmidt reaction of 2-(4-methoxyphenyl)-2-azabicyclo[2.2.2]octan-5-one, which bears an unsubstituted ethano bridge, giving rise to a mixture of 3,6-and 2,6-diazabicyclo[3.2.2]nonanones in modest yields.^24^ The ratios of the migration products are affected by the presence of ring and bridge substituents^25^ and influenced by reaction conditions, including the acid used and the acid concentration, as demonstrated in acyclic systems.^26^

A promising yet underutilized strategy for controlling the regioselectivity of nitrogen insertion was reported by Lattes^27,28^ and developed by Aubé^29−32^ and proceeds via the photochemical rearrangement of oxaziridines (Scheme 1). The oxaziridine intermediate could be formed selectively via a combination of diaxial and chiral amine-controlled imine oxidation and upon photochemical activation by ultraviolet light would rearrange under stereoelectronic control to one lactam with high selectivity.^33^ For α-substituted ketones, a product distribution complementary to that from the Schmidt reaction was possible.^27^

Photochemistry can be successfully implemented for large-scale manufacture, as exemplified by the industrial-scale Toray process, although its widespread acceptance as a practical method has been hampered by a lack of reliably scalable processes.^34^ Continuous-flow photochemical reactors have been developed to address this problem, though their design has tended toward the use of higher-energy, more intense light sources within increasingly complex reactors.^35^ A practical reactor developed by GlaxoSmithKline and the Booker-Milburn group utilizing a fluoropolymer (FEP)-wrapped high-power UV source was used to demonstrate a range of cycloaddition reactions.^36^ This system was successfully utilized for the scale-up of a chiral bicyclic lactam using photochemical oxaziridine rearrangement chemistry, with high rearrangement selectivity at moderate reaction conversions.^37^ More recently, higher-throughput reactors based on kilowatt UV light sources such as the Firefly parallel tube flow reactor^38^ and continuous Taylor vortex reactor^39^ have been developed and have achieved impressive productivities of multiple kilograms per day.

Reaction activation by light can be an environmentally benign process. As the majority of photochemical reactions occur only within a short distance from the solution interface exposed to radiation (see the Supporting Information), efforts toward more efficient and sustainable energy use have seen the continued development of photoreactors with micrometer-sized channels.^34,40−42^ These are highly effective at utilizing incoming radiation and allow the use of more efficient low-power lamps, light-emitting diodes, or natural light sources, which in turn reduce reactor complexity and offer advantages in safety. Achieving the productivity of macro-flow systems then requires only extending the reaction time or numbering-up multiple low-cost devices.

Herein we describe our work toward the scale-up of chiral bicyclic homopiperazines **1**. Continuous-flow chemistry was used to improve the safety and outcome of the Schmidt rearrangement over the batch method. The important progress described here is the first use of a photochemical oxaziridine rearrangement to achieve the scale-up of a homopiperazine. An efficient and inexpensive microreactor was designed to achieve scalable photochemistry using continuous-flow.

## Results and Discussion

**Scheme 2:**
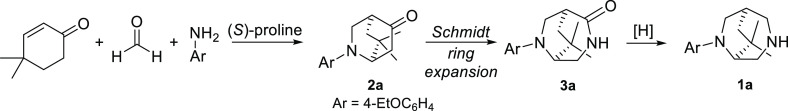
Initial Synthetic Route to Bicyclic Homopiperazine **1a**

Our initial approach to the synthesis of homopiperazine **1a** was based on the Schmidt reaction of 2-azabicyclo[2.2.2]octanone **2a** (Scheme 2), which was readily available in high enantiomeric purity on a multigram scale using (*S*)-proline-catalyzed aza-Diels–Alder batch chemistry.^43^ Subsequent amide reduction of **3a** would furnish the required homopiperazine **1a** which could undergo further modification to homopiperazines **1b** and **1c.**

Initial experiments were carried out on a 0.5–2.5 g (1–9 mmol) scale of ketones **2** in batch mode. Upon treatment of **2a** (Ar = 4-EtOC_6_H_4_) with sodium azide in concentrated sulfuric acid, lactam products from methylene migration (**3a**) and bridgehead methine migration (**4a**) were obtained, albeit in low overall yield (Table 1, entry 1). Similarly modest yields were obtained from a range of *N*-aryl-substituted ketones **2b**–**e**, but in each case selectivity for the desired lactam **3** was moderate or non-existent (Table 1, entry 2–5). The use of polyphosphoric acid (115% H_3_PO_4_ grade) as the solvent and proton source gave similar results (entry 7). When concentrated acids were used, strong magnetic stirring was required to ensure efficient mixing. Our attempts to improve the reaction outcome by employing Lewis acidic conditions (TiCl_4_; entry 6) or using trifluoroacetic acid (entry 8) were unsuccessful. Accurate control of the reaction time was challenging, as quenching the reaction required a lengthy (2 h) neutralization of the concentrated acid solvent. Extending the reaction time from 2.5 to 4 h led to a complex reaction mixture and poor recovery of organic material. Increasing the reaction scale (4-fold in **2b**; entry 9) while attempting to maintain a 2.5 h reaction duration gave a higher conversion to lactam products, but the selectivity for the desired lactam **3** was diminished, likely affected during the 3 h required to fully quench the reaction.

**Table 1:**
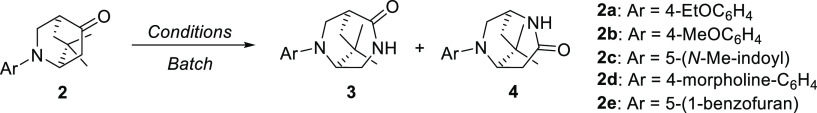
Ring Expansion via Schmidt and Beckmann Rearrangement in Batch

			yield (%)		
entry	substrate	conditions^a^	**3**	**4**	**3**:**4**
1	**2a**	NaN_3_, H_2_SO_4_, 0 °C	26	15	1.7:1
2	**2b**	NaN_3_, H_2_SO_4_, 0 °C	10	8	1.3:1
3	**2c**	NaN_3_, H_2_SO_4_, 0 °C	20	12	1.6:1
4	**2d**	NaN_3_, H_2_SO_4_, 0 °C	27	17	1.6:1
5	**2e**	NaN_3_, H_2_SO_4_, 0 °C	15	17	0.9:1
6	**2e**	NaN_3_, TiCl_4_, CH_2_Cl_2_, 0 °C^b^	–c	–c	–
7	**2a**	NaN_3_, PPA, 0 °C^d^	19	11	1.7:1
8	**2a**	NaN_3_, TFA, AcOH, rt^e^	–c	–c	–
9	**2b**	NaN_3_, H_2_SO_4_, 0 °C^f^	24	31	0.8:1
10	**2a**	NH_2_OH·HCl, MeOH/CH_2_Cl_2_ (5:3), rt, 3 h, then SOCl_2_, THF, 0 °C^g^	0	39	0:1
11	**2a**	NH_2_OH·HCl, Et_3_N, MeOH/CH_2_Cl_2_ (5:3), 0 °C, 3 h, then SOCl_2_, THF, 0 °C^g^	12	19	0.6:1

a 2.3 equiv of NaN_3_, 2.5 h, unless otherwise stated. Scale: 1–9 mmol of **2**.

b 2.5 equiv of TiCl_4_.

c No reaction after 16 h.

d PPA = polyphosphoric acid.

e 1.5 equiv of trifluoroacetic acid.

f Scale: 36 mmol of **2b**.

g 1.3 equiv of NH_2_OH·HCl.

The related Beckman rearrangement usually gives a product distribution determined by the stereochemistry of the intermediate oxime.^44,45^ Treatment of **2a** with hydroxylamine-*O*-sulfonic acid in formic or acetic acid^23^ led only to decomposition products. Stepwise formation of the intermediate oxime with hydroxylamine under thermodynamic conditions (Table 1, entry 10) (stereoisomer ratio of 9.1:1 by ^1^H NMR) followed by rearrangement yielded exclusively lactam **4a**. Conditions were optimized for formation of the kinetic oxime (entry 11) (stereoisomer ratio of 0.32:1 by ^1^H NMR), but the rearrangement preference for lactam **4a** could not be overcome.

We were at this time approaching our maximum scale limit for the generation of hydrazoic acid in our laboratory. The additional hazards presented by the large volumes of concentrated acid necessary for the Schmidt rearrangement and the impractical nature of the lengthy neutralization procedure prompted us to investigate scaling the Schmidt reaction in continuous-flow. We envisaged that the microreactor system using MsOH–DME^13^ in combination with tetrabutylammonium azide (TBAA) as a soluble azide source, as recently demonstrated by Jia,^14^ could provide a practical way to overcome these issues.

**Figure 2:**
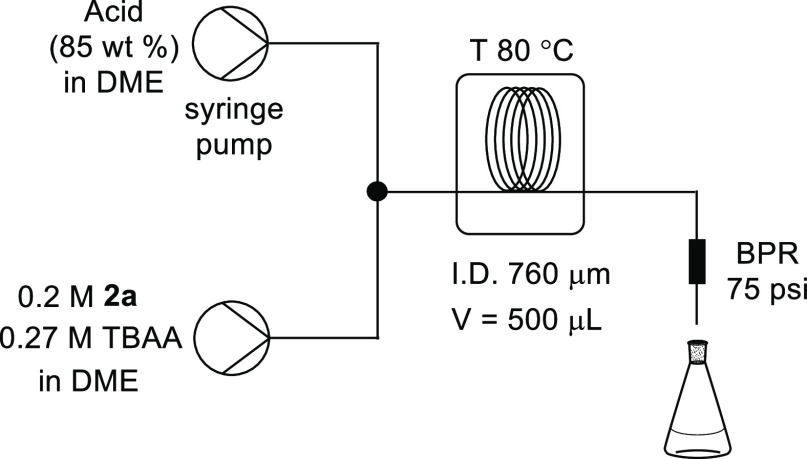
Continuous-flow Schmidt microreactor setup.

With a similar microreactor system (Figure 2) under the optimized conditions (0.20 M ketone, 0.27 M TBAA, 67% MsOH; *t*_R_ = 5 min, 80 °C), bicyclic ketone **2a** gave only trace rearrangement products. Increasing the average residence time to 6.7 min (Table 2, entry 1) improved the reaction conversion, but a modest preference for the undesired lactam **4a** was observed. The acid strength, in particular the use of dilute sulfuric acid, has been shown to have an important influence on the Schmidt reaction of alkyl cyclopropyl ketones, reversing the product distribution seen with the concentrated acid.^26^ No additional difficulties were encountered using sulfuric acid in the microreactor, though to facilitate its handling the concentrated acid required dilution to 85 wt % with DME to reduce its viscosity. The relative flow rates of the ketone/azide and acid solutions were then varied to achieve the acid concentrations stated (Table 2, entries 3–6) while a constant average residence time (8.6 min) was maintained.^46^

**Table 2:**
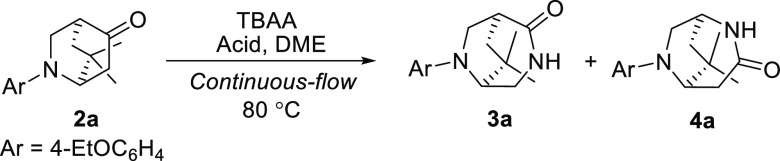
Schmidt Rearrangement in Continuous-Flow^a^

				yield (%)		
entry	*c*_**2a**_ (M)	acid, wt %	*t*_R_ (min)	**3a**	**4a**	**3a**:**4a**
1	0.20	MsOH, 67	6.7	8	10	0.8:1
2	0.20	H_2_SO_4_, 66	6.7	16	17	1:1.1
3	0.20	H_2_SO_4_, 48	8.6	14	12	1.2:1
4	0.20	H_2_SO_4_, 59	8.6	28	26	1.1:1
5	0.20	H_2_SO_4_, 69	8.6	29	26	1.1:1
6	0.20	H_2_SO_4_, 77	8.6	40	39	1:1
7	0.31	H_2_SO_4_, 77	8.6	41	37	1.1:1

a TBAA = tetrabutylammonium azide (1.35 equiv). *c*_**2a**_ = concentration of **2a**. *t*_R_ = average retention time.

We would like to highlight a number of safety precautions taken here. During operation, the micromixer and reactor were submerged in water baths containing 5 wt % NaHCO_3_ in order that any leak in the system could be quickly identified by the evolution of gas. Excess HN_3_ in the outflow stream was neutralized upon collection into a vessel containing 2 M NaOH/ice (1:1), and operation of the microreactor was carried out behind a blast shield. Furthermore, our reaction-scale limit was set such that in the event of total release of all azide as hydrazoic acid and fume cupboard failure, the concentration within the laboratory could not reach predefined limits.

Although using sulfuric acid had little effect on the product ratio at the elevated temperatures in the microreactor, increasing the acid concentration significantly improved the reaction conversions and overall yields. However, in order to achieve the increased acidity in this system, the relative flow rate of ketone/azide to acid solution had to be reduced significantly, resulting in a productivity of only 0.04 mmol h^–1^ for **3a** (entry 6). Increasing the ketone/azide concentration to the upper solubility limit of the ketone in DME (0.31 M; entry 7) allowed only a marginal improvement in productivity to 0.06 mmol h^–1^.

Although utilizing the continuous-flow format allowed us to improve the efficiency of the Schmidt reaction over our batch process and, importantly, provided an effective way of mitigating hazards associated with the generation of large amounts of HN_3_, we were nevertheless dissatisfied with the low productivity and the equivalent of waste lactam that needed to be discarded through poor regioselectivity for the nitrogen insertion. While further optimization of the productivity of the system may be possible, reaction regioselectivity would likely remain an issue.

**Scheme 3:**
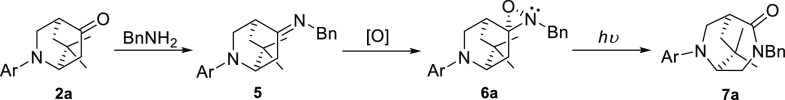
Proposed Oxaziridine Rearrangement to **7a**

We were attracted by the oxaziridine rearrangement chemistry of Lattes and Aubé as a potential solution. The rearrangement of oxaziridines to amides proceeds under stereoelectronic control, with generally good regioselectivity for the amide where the carbon substituent anti to the nitrogen lone pair migrates.^33^ Chiral amines have previously been used with prochiral substrates in order to exert facial control of the imine oxidation. For ketone **2a**, we hypothesized that condensation with a simple amine under equilibrating conditions would provide predominantly imine **5** (Scheme 3) because of the steric influence of the bridgehead. Benzylamine was therefore chosen as the amine on the basis of its low cost, ready availability, and ease of removal by hydrogenolysis. Because of the conformational restriction imposed by the bridge, oxidation could then occur from the less hindered (exo) face to give desired oxaziridine diastereoisomer **6a**.^31^ Photochemical rearrangement would then provide benzyl-protected lactam **7a**.^33^

**Scheme 4:**
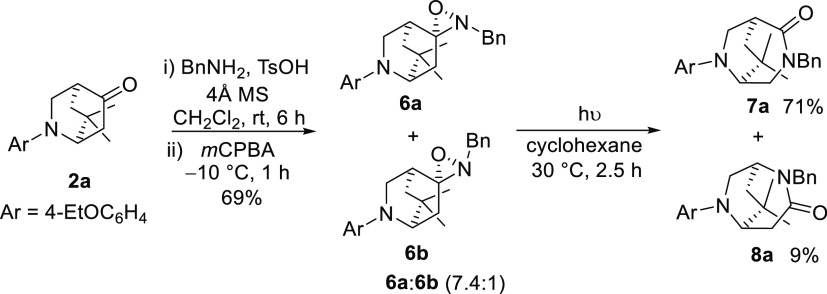
Oxaziridine Rearrangement in Batch

Pleasingly, gram-scale (4–18 mmol) batch condensation of ketone **2a** with benzylamine in the presence of catalytic 4-toluenesulfonic acid resulted in complete imine formation within 6 h at room temperature (Scheme 4). After filtration, addition of several oxidants, including peracetic acid and Oxone, gave complex reaction mixtures. *m*-Chloroperbenzoic acid proved to be more selective, providing diastereomeric oxaziridines **6a** and **6b** in ratios of 4:1 at room temperature to 9:1 at −10 °C. Oxaziridine **6b** arises from oxidation of the minor imine stereoisomer likely formed by isomerization during the imine workup and/or oxidation steps. Its formation could be minimized by prompt filtration of the imine solution under an atmosphere of dry nitrogen gas and by performing the oxidation below room temperature.

The oxaziridines formed were crystalline solids that were conformationally stable at room temperature and isolated by filtration through a short silica plug. Attempts to separate them by crystallization or chromatography on a preparative scale were unsuccessful. Pleasingly, upon ultraviolet irradiation of the diastereomeric mixture of **6** (**6a**:**6b** = 7.4:1) in degassed cyclohexane in a small-scale batch reactor, rearrangement gave ring-expanded lactams **7a** and **8a** in high overall yield with the oxaziridine isomer ratio translating well to the lactam ratio (**7a**:**8a**, 7.9:1). Cyclohexane, diethyl ether, and acetonitrile were tested as solvents for the rearrangement. Although each gave the desired rearrangement products, in acetonitrile minor side reactions could be observed (5–10%) leading to partial recovery of ketone **2a**. The low boiling point of diethyl ether led to some solvent loss inside the batch reactor, and it was rejected as a suitable solvent for this reason. Cyclohexane gave the cleanest conversion to lactam products and was taken forward as the preferred solvent.

The imine formation and oxidation steps were optimized to provide oxaziridine **6a**, and our attention then turned toward scale-up of the photochemical step. We expected that a continuous-flow microreactor system could provide a simple and inexpensive solution. A low-pressure lamp efficient at conversion of input power to UV C radiation could be used, without the need for the specialized UV-transparent (quartz) immersion-well glassware and forced coolant as required by higher-pressure lamps, which emit strongly in the infrared. Furthermore, opportunity for exposure to the operator of intense UV radiation would be minimized.

**Figure 3:**
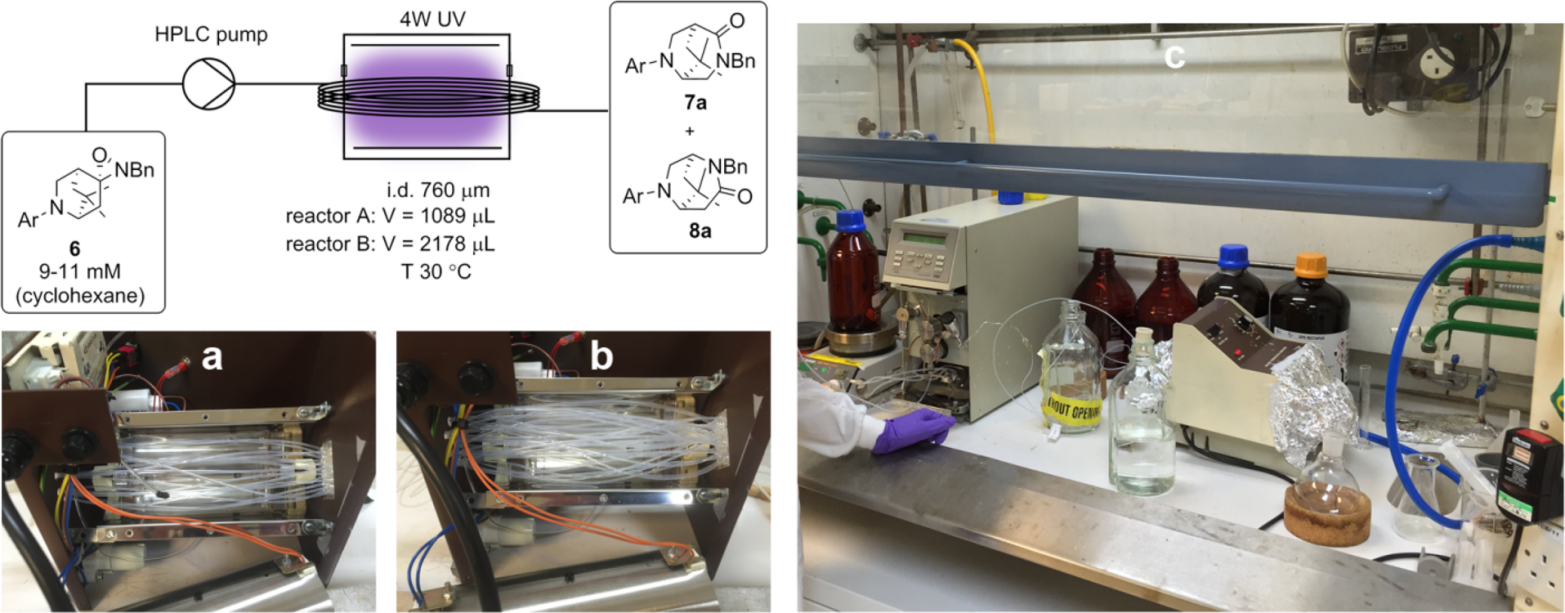
Continuous-flow photochemical microreactor setup. (a, b) View inside reactor with the cylindrical metal reflector removed: (a) reactor A, 16-pass and (b) reactor B, 32-pass. (c) Full setup inside a 1.8 m fume cupboard.

A parallel tube flow microreactor (Figure 3) was constructed as follows. The setup consisted of an HPLC pump, a 15 m length of UV-transparent perfuoroalkoxy (PFA) tubing (760 μm, i.d.), and a collection vessel arranged in series. The PFA tubing was threaded several times through the batch photochemical microreactor, comprising a 4 W UV C bulb inside a cylindrical reflector, such that serial sections of tubing could be exposed to UV radiation (reactor A, 16-pass, *V* = 1089 μL, 2.40 m irradiated length; full details are provided in the Supporting Information).

Cyclohexane solutions of oxaziridine **6** (6:1 to 9:1 by ^1^H NMR) were thoroughly degassed with argon, pumped through the photochemical reactor, and collected at the outlet. Initial experiments were conducted at close to the upper solubility limit of **6** in cyclohexane (11 mM, 4 g L^–1^). High reaction conversions were observed at a flow rate of 0.30 mL min^–1^ (Table 3, entry 1). However, in an identical run with these parameters, the less soluble lactam products began to precipitate inside the reactor tubing, reducing the reaction conversion. Lowering the oxaziridine concentration to 9 mM (3.3 g L^–1^) was sufficient to completely overcome this issue. The flow rate could then be increased to an optimum value of 0.75 mL min^–1^ while maintaining the reaction conversion at above 95% (entry 2).

**Table 3:**
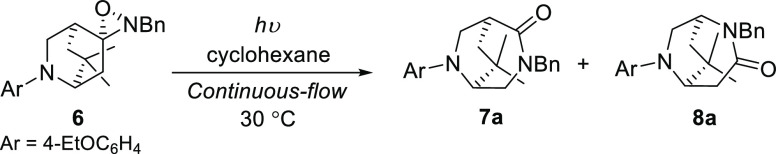
Oxaziridine Rearrangement in Continuous-Flow

						yield (%)				
entry	reactor	*c* (mM)^a^	flow rate (mL min^–1^)	*t*_R_ (min)^b^	*t* (h)^c^	**7a**	**8a**	**7a**:**8a**	productivity (mmol h^–1^)^d^	STY (mmol h^–1^ mL^–1^)^e^
1	A	11	0.30	3.6	17	71	12	5.9:1	0.14	0.12
2	A	9	0.75	1.5	26	82	11	7.5:1	0.32	0.29
3	A	9	0.75	1.5	42	84	11	7.6:1	0.33	0.30
4	B	9	1.20	1.8	8	85	10	8.5:1	0.53	0.24

a *c* = concentration.

b *t*_R_ = average retention time.

c *t* = time.

d Productivity = concentration × flow rate × yield × 60.

e STY is the space-time yield, given by STY = productivity/reactor volume.

Under these conditions, no maintenance of the flow reactor was required, and it could be operated continually for an extended period (42 h) without decay of the reaction conversion (Table 3, entry 3). Furthermore, the cyclohexane solvent used could be distilled and recycled through the reactor without loss of reaction performance.

In order to determine the maximum capability of this system, a modified reactor was tested. The length of tubing exposed to the UV lamp was doubled (reactor B, 32-pass, *V* = 2178 μL, 4.80 m irradiation length). With this design, the flow rate was increased until a drop in reaction conversion was observed. At a flow rate of 1.20 mL min^–1^, a productivity of 0.53 mmol h^–1^ (space-time yield = 0.24 mmol h^–1^ mL^–1^) for **7a** was possible (Table 3, entry 4). To benchmark the efficiency of this system against other flow reactors, our system employing a low-power (4 W) lamp compares favorably to the FEP-wrapped reactor design used for photochemical rearrangements to chiral lactams, which demonstrated a productivity of 24.9 mmol h^–1^ (space-time yield = 0.19 mmol h^–1^ mL^–1^) employing a medium-power (450 W) lamp.^47^ Although the present flow reactor was used only to demonstrate the gram-scale synthesis of **7a** (total amount synthesized: 17 g, 45 mmol), scaling to greater throughput should be straightforward by either extending the reaction time, numbering-up multiple flow reactors of this design, or translating the reaction to a larger-volume reactor.

**Scheme 5:**
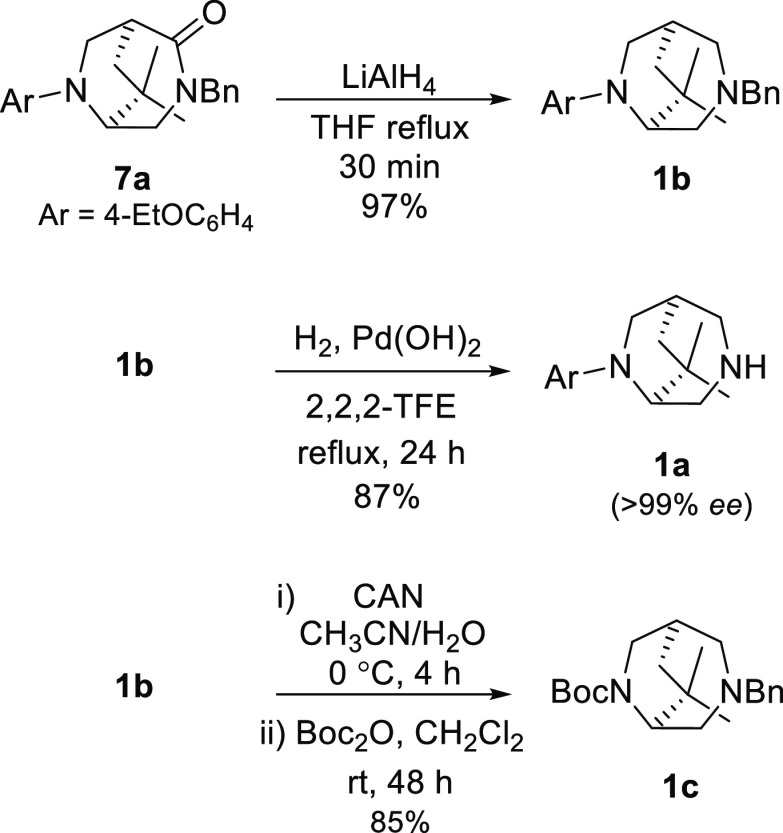
Reduction and Deprotection to Homopiperazines **1a**–**c**

Homopiperazinone **7a** was isolated by flash chromatography, and further chemistry was carried out on the gram scale in batch mode (Scheme 5). Reduction with LiAlH_4_ in THF gave versatile homopiperazine intermediate **1b** bearing orthogonal protecting groups. Differential deprotection was then possible. Catalytic hydrogenolysis to homopiperazine **1a** was achieved over palladium hydroxide using 2,2,2-trifluoroethanol (2,2,2-TFE) as the solvent,^48^ which avoided the formation of *N*-alkylated byproducts observed when the reaction was performed in ethanol. The optical purity of **1a** was determined by derivatization and analysis on a chiral stationary phase (>99% ee). The ethoxyphenyl group of **1b** could be removed efficiently by oxidation with cerium ammonium nitrate. During the reaction, complete monodeprotection to the *NH*-homopiperazine was observed, but isolation of the basic homopiperazine from the reaction mixture proved to be challenging. In-situ *tert*-butoxycarbonyl protection assisted isolation to provide homopiperazine **1c**.

## Conclusion

A key ring-expansion step for the scale-up of a chiral bicyclic homopiperazine via Schmidt rearrangement and Lattes–Aubé chemistry was investigated. Adopting continuous-flow allowed access to preferential conditions for the Schmidt rearrangement and an improved safety profile over the batch process, although the productivity and selectivity for the required homopiperazine isomer remained moderate. Oxaziridine rearrangement chemistry gave greater selectivity for the ring-expansion step under milder reaction conditions. Conversion of the photochemical rearrangement step to continuous-flow in an efficient flow reactor utilizing a low-power source allowed the multigram scale-up of the otherwise difficult to access homopiperazines **1a**–**c**. Future advances in UV-LED or solar energy capture technology will likely further improve the potential for more environmentally benign methods for photochemical synthesis and encourage the development of efficient and scalable routes to other pharmaceutically useful nitrogen-containing heterocycles.

## Experimental Section

### (1*R*,4*S*)-2-(4-Ethoxyphenyl)-7,7-dimethyl-2-azabicyclo[2.2.2]octan-5-one (**2a**)

This compound was prepared following a modified literature procedure.^43^ To a solution of *p*-phenetidine (10 mL, 84.5 mmol), 4,4-dimethyl-2-cylohexen-1-one (11.12 mL, 77.5 mmol), and l-proline (2.43 g, 21.1 mmol) in DMSO (200 mL) was added dropwise 37% aqueous formaldehyde (5.25 mL, 70.4 mmol). The reaction mixture was stirred at room temperature for 16 h and then diluted with EtOAc (250 mL) and water (250 mL). The layers were separated, and the aqueous phase was extracted with EtOAc (2 × 150 mL). The combined organic phases were washed with water (5 × 150 mL) and brine (100 mL), dried (MgSO_4_), and concentrated under vacuum. Recrystallization (methanol/water) afforded **2a** (13.66 g, 71%) as a light-brown solid. [α]_D_^12^ = −85.9 (*c* = 1.7, CHCl_3_). ^1^H NMR (500 MHz, CDCl_3_) δ 6.90–6.76 (m, 2H), 6.69–6.50 (m, 2H), 3.97 (q, *J* = 7.0 Hz, 2H), 3.75 (t, *J* = 2.7 Hz, 1H), 3.53–3.40 (m, 2H), 2.68 (dd, *J* = 18.9, 2.1 Hz, 1H), 2.62 (p, *J* = 2.8 Hz, 1H), 2.47 (dd, *J* = 18.9, 3.2 Hz, 1H), 1.77 (d, *J* = 3.0 Hz, 2H), 1.38 (t, *J* = 7.0 Hz, 3H), 1.09 (s, 3H), 1.08 (s, 3H). ^13^C NMR (75 MHz, CDCl_3_) δ 214.0, 150.5, 143.1, 116.1 (2C), 111.9 (2C), 64.3, 58.2, 47.8, 45.9, 41.2, 38.8, 36.0, 30.1, 28.8, 15.2. HRMS calcd for C_17_H_24_NO_2_ [M + H]^+^ 274.1807, found 274.1801.

### Schmidt Rearrangement of **2a** in Continuous-Flow (Table 2, Entry 6)

A solution of H_2_SO_4_ (85% w/w) in 1,2-dimethoxyethane (flow rate: 3.00 mL hr^–1^) and a binary solution of **2a** (0.20 M) and tetrabutylammonium azide (0.27 M) in 1,2-dimethoxyethane (0.50 mL hr^–1^) were fed via syringe pump through the microreactor coil (760 μm i.d., 500 μL volume) immersed in a water bath at 80 °C. After steady-state conditions were reached (17 min), the outflow was collected for 3.5 h. The material in the collection vessel was brought to pH 9 with 2 M NaOH and then extracted with CH_2_Cl_2_ (3 × 30 mL). The combined organic phases were washed with brine (30 mL), dried (MgSO_4_), and concentrated under vacuum. Column chromatography (ethyl acetate/petroleum ether) afforded (1*S*,5*S*)-6-(4-ethoxyphenyl)-9,9-dimethyl-3,6-diazabicyclo[3.2.2]nonan-2-one (**3a**) (40 mg, 40%) and (1*S*,5*R*)-6-(4-ethoxyphenyl)-9,9-dimethyl-2,6-diazabicyclo[3.2.2]nonan-3-one (**4a**) (39 mg, 39%).

#### (1*S*,5*S*)-6-(4-Ethoxyphenyl)-9,9-dimethyl-3,6-diazabicyclo[3.2.2]nonan-2-one (**3a**)

^1^H NMR (500 MHz, CDCl_3_) δ 6.88–6.82 (m, 2H), 6.68–6.61 (m, 2H), 5.48 (s, 1H), 3.97 (q, *J* = 7.0 Hz, 2H), 3.67 (s, 1H), 3.55 (dt, *J* = 10.8, 1.7 Hz, 1H), 3.49 (dt, *J* = 12.6, 2.8 Hz, 1H), 3.44 (ddd, *J* = 12.6, 3.1, 1.8 Hz, 1H), 3.38 (dd, *J* = 10.8, 4.9 Hz, 1H), 2.93–2.87 (m, 1H), 1.95 (dt, *J* = 14.3, 1.8 Hz, 1H), 1.65 (dd, *J* = 14.3, 5.9 Hz, 1H), 1.38 (t, *J* = 7.0 Hz, 3H), 1.19 (s, 3H), 1.10 (s, 3H). ^13^C NMR (75 MHz, CDCl_3_) δ 177.9, 150.9, 142.9, 116.1 (2C), 112.3 (2C), 64.3, 60.1, 46.5, 43.6, 41.4, 37.2, 35.5, 32.0, 28.6, 15.2. HRMS calcd for C_17_H_25_N_2_O_2_ [M + H]^+^ 289.1916, found 289.1911.

#### (1*S*,5*R*)-6-(4-Ethoxyphenyl)-9,9-dimethyl-2,6-diazabicyclo[3.2.2]nonan-3-one (**4a**)

^1^H NMR (500 MHz, CDCl_3_) δ 7.19 (t, *J* = 9.6 Hz, 1H), 6.91–6.82 (m, 2H), 6.68–6.51 (m, 2H), 3.97 (q, *J* = 7.0 Hz, 2H), 3.65–3.54 (m, 1H), 3.53–3.43 (m, 3H), 2.90–2.72 (m, 2H), 1.92 (d, *J* = 14.2 Hz, 1H), 1.69 (dd, *J* = 14.3, 4.9 Hz, 1H), 1.37 (t, *J* = 7.0 Hz, 3H), 1.16 (s, 3H), 1.06 (s, 3H). ^13^C NMR (75 MHz, CDCl_3_) δ 174.7, 150.8, 143.1, 116.1 (2C), 112.3 (2C), 64.3, 57.4, 51.4, 47.2, 41.8, 36.9, 36.2, 32.0, 29.4, 15.2. HRMS calcd for C_17_H_24_N_2_O_2_Na [M + Na]^+^ 311.1735, found 311.1721.

### (1*S*,2*R*,4*R*)-2′-Benzyl-5-(4-ethoxyphenyl)-8,8-dimethyl-5-azaspiro[bicyclo[2.2.2]octane-2,3′-[1,2]oxaziridine] (**6**)

To a mixture of **2a** (5.00 g, 18.29 mmol) and 4 Å molecular sieves (10 g) in anhydrous CH_2_Cl_2_ (35 mL) was added dropwise benzylamine (2.00 mL, 18.29 mmol) followed by TsOH·H_2_O (7 mg, 0.03 mmol), and the mixture was allowed to stand for 6 h at room temperature. The reaction mixture was filtered promptly through glass wool to remove the molecular sieves using anhydrous CH_2_Cl_2_ (25 mL) under a stream of dry nitrogen gas. The solution was then cooled to −10 °C, and a solution of *m*CPBA (≤77 wt %, 4.92 g, 21.95 mmol) in anhydrous CH_2_Cl_2_ (40 mL) was added dropwise under rapid stirring. After the addition, the reaction mixture was maintained at −10 °C for 1 h and then allowed to warm to room temperature. The reaction mixture was cooled to 0 °C and then quenched by the addition of saturated Na_2_S_2_O_3_/saturated NaHCO_3_ (1:1, 10 mL) followed by water (40 mL). The phases were separated, and the organic phase was extracted with CH_2_Cl_2_ (2 × 50 mL). The combined organic phases were washed successively with 10% aqueous NaHCO_3_ (50 mL) and brine (30 mL), dried (MgSO_4_), and concentrated under vacuum. Column chromatography (ethyl acetate/cyclohexane) afforded **6** (4.79 g, 69%) as a mixture of diastereoisomers (7.4:1).

#### (1*S*,2*R*,2′*s*,4*R*)-2′-Benzyl-5-(4-ethoxyphenyl)-8,8-dimethyl-5-azaspiro[bicyclo[2.2.2] octane-2,3′-[1,2]oxaziridine] (**6a**, Major Isomer)

^1^H NMR (500 MHz, CDCl_3_) δ 7.43 (d, *J* = 7.3 Hz, 2H), 7.37 (t, *J* = 7.3 Hz, 2H), 7.31 (t, *J* = 7.3 Hz, 1H), 6.85 (d, *J* = 9.1 Hz, 2H), 6.60 (d, *J* = 9.1 Hz, 2H), 3.98 (q, *J* = 6.9 Hz, 2H), 3.90 (app q, *J* = 15.1 Hz, 2H), 3.58 (t, *J* = 2.5 Hz, 1H), 3.51 (dt, *J* = 10.9, 2.5 Hz, 1H), 3.26 (dt, *J* = 9.8, 2.2 Hz, 1H), 2.61 (dd, *J* = 15.5, 2.2 Hz, 1H), 2.26 (dd, *J* = 15.5, 3.5 Hz, 1H), 1.67 (t, *J* = 2.5 Hz, 1H), 1.60–1.57 (m, 2H), 1.39 (t, *J* = 6.9 Hz, 3H), 0.98 (s, 3H), 0.89 (s, 3H). ^13^C NMR (125 MHz, CDCl_3_) δ 150.0, 143.4, 136.4, 128.8 (2C), 128.7 (2C), 127.8, 116.2 (2C), 111.5 (2C), 86.2, 64.4, 59.4, 55.9, 46.2, 38.5, 37.1, 34.9, 30.1, 29.0, 27.9, 15.2. HRMS calculated for C_24_H_31_N_2_O_2_ [M + H]^+^ 379.2386, found 379.2370.

### Photochemical Rearrangement in Continuous-Flow (Table 3, Entry 3)

A solution of **6** (6.11 g, 16.10 mmol) in cyclohexane (1.875 L, *c* = 3.26 g L^–1^) was pumped through PFA tubing (15.00 m total length, 760 μm i.d., 6800 μL volume), including a section irradiated with a 4 W UV C bulb (λ_max_ = 254 nm) (2.40 m length, 1089 μL volume, 760 μm i.d.), for 41.5 h via an HPLC pump (flow rate 0.75 mL min^–1^, average residence time 87 s) and collected at the outlet. The solvent was removed under vacuum and recycled. Column chromatography (ethyl acetate/cyclohexane) gave (1*S*,5*S*)-3-benzyl-6-(4-ethoxyphenyl)-9,9-dimethyl-3,6-diazabicyclo[3.2.2]nonan-2-one (**7a**) (5.11 g, 84%) and (1*S*,5*R*)-2-benzyl-6-(4-ethoxyphenyl)-9,9-dimethyl-2,6-diazabicyclo[3.2.2]nonan-3-one (**8a**) (660 mg, 11%).

#### (1*S*,5*S*)-3-Benzyl-6-(4-ethoxyphenyl)-9,9-dimethyl-3,6-diazabicyclo[3.2.2]nonan-2-one (**7a**)

^1^H NMR (500 MHz, CDCl_3_) δ 7.32–7.24 (m, 5H), 6.86 (d, *J* = 8.8 Hz, 2H), 6.65 (d, *J* = 9.1 Hz, 2H), 4.82 (d, *J* = 14.5 Hz, 1H), 4.26 (d, *J* = 14.5 Hz, 1H), 3.99 (q, *J* = 6.9 Hz, 2H), 3.62 (t, *J* = 3.2 Hz, 1H), 3.58 (d, *J* = 10.7 Hz, 1H), 3.41 (dd, *J* = 5.4, 10.7 Hz, 1H), 3.37 (d, *J* = 3.5 Hz, 2H), 3.17–3.15 (m, 1H), 1.95 (d, *J* = 14.2 Hz, 1H), 1.68 (dd, *J* = 5.7, 14.2 Hz, 1H), 1.39 (t, *J* = 6.9 Hz, 3H), 1.07 (s, 3H), 0.94 (s, 3H). ^13^C NMR (125 MHz, CDCl_3_) δ 174.9, 150.7, 142.8, 136.9, 128.6 (2C), 128.3 (2C), 127.5, 115.9 (2C), 112.3 (2C), 64.1, 60.6, 50.6, 49.6, 46.6, 42.1, 37.2, 35.1, 31.6, 27.9, 15.8. HRMS calcd for C_24_H_31_N_2_O_2_ [M + H]^+^ 379.2386, found 379.2364.

#### (1*S*,5*R*)-2-Benzyl-6-(4-ethoxyphenyl)-9,9-dimethyl-2,6-diazabicyclo[3.2.2]nonan-3-one (**8a**)

^1^H NMR (500 MHz, CDCl_3_) δ 7.36–7.30 (m, 5H), 6.85 (d, *J* = 9.1 Hz, 2H), 6.63 (d, *J* = 9.1 Hz, 2H), 4.80 (d, *J* = 14.8 Hz, 1H), 4.53 (d, *J* = 14.8 Hz, 1H), 3.99 (q, *J* = 7.3 Hz, 2H), 3.66–3.64 (m, 1H), 3.51 (t, *J* = 3.8 Hz, 1H), 3.43–3.39 (m, 1H), 3.31 (d, *J* = 11.4 Hz, 1H), 2.93 (dq, *J* = 4.1, 12.9 Hz, 2H), 1.70 (dt, *J* = 2.2, 14.2 Hz, 1H), 1.55 (dd, *J* = 4.7, 14.5 Hz, 1H), 1.39 (t, *J* = 6.9 Hz, 3H), 1.11 (s, 3H), 1.07 (s, 3H). ^13^C NMR (125 MHz, CDCl_3_) δ 171.3, 150.9, 142.9, 137.5, 128.7 (2C), 128.4 (2C), 127.6, 115.9 (2C), 112.9 (2C), 64.1, 58.4, 52.6, 51.5, 51.0, 39.8, 37.6, 35.4, 31.5, 28.6, 15.1. HRMS calcd for C_24_H_31_N_2_O_2_ [M + H]^+^ 379.2386, found 379.2368.

## Supporting Information Available

Experimental procedures, including setup and operation of the Schmidt microreactor and the photochemical flow reactor, and characterization data for compounds **1a**–**c**, **2a**, and **3a**–**8a** (PDF)

## Abbreviations

LOX: lysyl oxidase
*t*_R_: average residence time
DME: 1,2-dimethoxyethane
MsOH: methanesulfonic acid
TBAA: tetrabutylammonium azide
TsOH·H_2_O: *p*-toluenesulfonic acid monohydrate
Bn: benzyl
PFA: perfluoroalkoxy
UV: ultraviolet
STY: space-time yield
THF: tetrahydrofuran
Boc: *tert*-butoxycarbonyl
CAN: cerium ammonium nitrate
