# Supplementary material for: Toward the Scale-Up of a Bicyclic Homopiperazine via Schmidt Rearrangement and Photochemical Oxaziridine Rearrangement in Continuous-Flow
Source: Org Process Res Dev. 2020 Oct 15;25(1):148–56. doi: 10.1021/acs.oprd.0c00361 (PMC7928940; doi:10.1021/acs.oprd.0c00361)
Supplement: Supplementary file 1 — op0c00361_si_001.pdf [file op0c00361_si_001.pdf]

Toward the Scale-up of a Bicyclic Homopiperazine via Schmidt Rearrangement and Photochemical Oxaziridine Rearrangement in Continuous-Flow

Michael Brown,[*,a,b] *Mohammed Aljarah,*[a,b] *Hannah Asiki,*[b] *Leo M. H. Leung,*[a,b] *Deborah A. Smithen,*[a,b] *Natalie Miller,* [b] *Gabor Nemeth,*[b] *Lawrence Davies,*[b] *Dan Niculescu-Duvaz,*[a,b] *Alfonso Zambon,*[b] *Caroline Springer* [a,b]

a. Drug Discovery Unit, Cancer Research UK Manchester Institute, University of Manchester, Alderley Park, Macclesfield, SK10 4TG, United Kingdom

b. Cancer Research UK Centre for Cancer Therapeutics, The Institute of Cancer Research, 15 Cotswold Road, London, SM2 5NG, United Kingdom

Supporting information

Contents

Beer-Lambert-Bouguer Law …………………………………………………………………………………………..3

General………..4

Preparation of Ketone 2a………………………………………………………………………………………. 5

Schmidt and Beckmann Rearrangements in Batch……………………………………………………6

Schmidt Rearrangement in Continuous-Flow …………………………………………………………8

Photochemical Reactor Setup…………………………………………………………………………… 11

Preparation of Oxaziridine 6 ……………………………………………………………………………...13

Photochemical Rearrangement in Continuous-Flow……………………………………………… 15

Reduction and Deprotection to Homopiperazines 1a-c ………………………………………… 17

Determination of Enantiomeric Excess of 1a ………………………………………………………… 19

References…………………………………………………………………………………………………… 21

Beer-Lambert-Bouguer law

$$A = \varepsilon c l$$

$$A = -log_{10}\frac{I}{I_0}$$

$$\frac{I}{I_0} = 10^{-\varepsilon c l}$$

Where: Absorption (*A*), Molar concentration (*c*), Path length (*l*), Molar absorption coefficient (*ε*), Transmission (*I/I₀*).

A typical transmission profile for a weakly absorbing n → σ* transition[1] is shown in Figure 1. Micro flow systems (<1 mm, i.d.) can absorb the majority of the incident radiation throughout the reaction medium, whereas in macro flow systems (>1 mm, i.d.) the majority of the reaction solution is screened from radiation by the material nearest the lamp. These systems are then dependent on mixing dynamics. To achieve efficient and scalable photolysis, reactor dimensions on the micrometer scale or less are preferred.

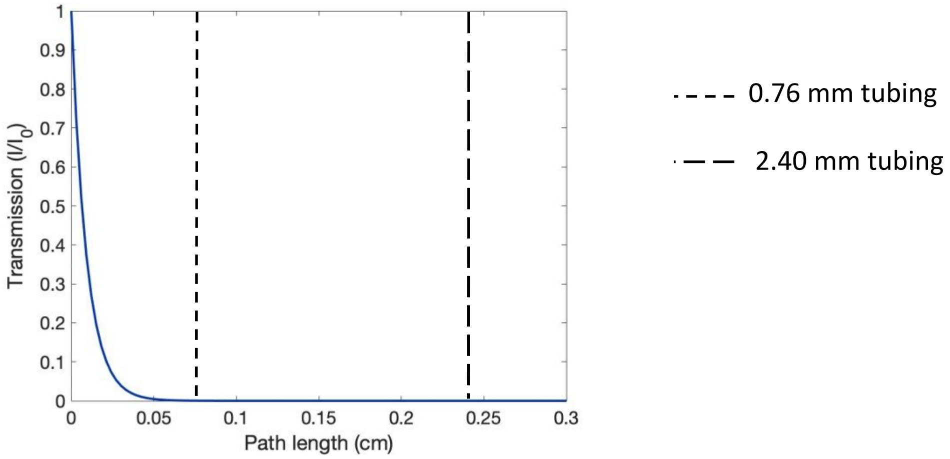

Figure S1. Example transmission profile for phenol (n → σ*): ε = 2340 L mol^{-1} cm^{-1}, λ = 271 nm, c = 0.02 M in cyclohexane.[2] Path length represents distance from the lamp. Dotted lines represent internal diameter of 760 µm tubing (this work) and 2.40 mm tubing (Cochran, J. E.; Waal, N. *Org. Process Res. Dev.* **2016**, *20* (8), 1533–1539).

General

All chemicals, reagents and anhydrous solvents were purchased from commercial sources and were used as received. Inhibitor-free, anhydrous 1,2-dimethoxyethane (DME) was purchased from Sigma Aldrich. Sulfuric acid (≥ 95%, d=1.83) was purchased from Fisher Scientific. *meta*-Chloroperbenzoic acid (≤ 77% wt %) was purchased from Sigma Aldrich and used without purification. HPLC grade cyclohexane was purchased from Fisher. Molecular sieves were dried in a microwave oven before use.

All microreactor parts and PFA High Purity tubing (1/16 × 0.30 × 50 ft) (internal diameter: 760 µm) were purchased from Kinesis (IDEX Health & Scientific). Two Razell A-99 syringe pumps were used for Schmidt reaction in continuous-flow after calibration over the range 0.71-70.0 $ml.hr^{-1}$. A SpectraSYSTEM P4000 Gradient Pump (Thermo Scientific) was used for continuous-flow photochemistry.

Thin layer chromatography analysis was performed using silica gel 60 F-254 thin layer plates. Flash chromatography was performed on a Biotage Isolera or Combiflash Rf+ UV-Vis flash purification system using pre-packed silica gel cartridges (Biotage) with HPLC grade solvents.

Specific rotations were measured on a Bellingham & Stanley ADP440 polarimeter with a path length of 0.5 dm. NMR spectra were recorded on Bruker Avance 300, 400 or 500 MHz spectrometer. ^{1}H NMR spectra were measured at 300, 400 or 500 MHz. ^{13}C NMR spectra were measured at 75, 100, 125 MHz using $CDCl_3$ or d_6-DMSO as the solvent and internal reference. Chemical shifts are expressed in parts per million (ppm). Coupling constants *J* are given in Hz. Multiplicity as follows: s = singlet, d = doublet, t = triplet, q = quartet, sext = sextet, sept = septet, m = multiplet, br = broad, app = apparent. Chemical shifts in brackets signify chemical shift arising from rotameric form.

HPLC and HRMS analyses were performed on an Agilent 1200 series HPLC (Agilent, Santa Clara, USA) and diode array detector coupled to a 6210 time-of-flight mass spectrometer with a multimode ESI source, or a Waters Acquity UPLC or I-Class UPLC with a diode array detector coupled to a Waters G2 QToF, SQD or QDa mass spectrometer fitted with a multimode ESI/APCI source.

Preparation of Ketone 2a

Ketones **2b**-**e** were prepared following the literature procedure.[3] Ketone **2a** was prepared following a modified literature procedure:

(1*R*,4*S*)-2-(4-Ethoxyphenyl)-7,7-dimethyl-2-azabicyclo[2.2.2]octan-5-one (**2a**)

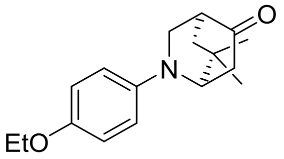

This compound was prepared following a modified literature procedure.[3] To a solution of *p*-phenetidine (10 mL, 84.5 mmol), 4,4-dimethyl-2-cylohexen-1-one (11.12 mL, 77.5 mmol) and L-proline (2.43 g, 21.1 mmol) in DMSO (200 mL) was added dropwise 37% aqueous formaldehyde (5.25 mL, 70.4 mmol). The reaction was stirred at room temperature for 16 h, then diluted with EtOAc (250 mL) and water (250 mL). The layers were separated and the aqueous phase extracted with EtOAc (2 × 150 mL). Combined organic phases were washed with water (5 × 150 mL), brine (100 mL), dried over anhydrous $MgSO_4$ and concentrated under vacuum. Recrystallization (methanol/water) afforded (1*R*,4*S*)-2-(4-ethoxyphenyl)-7,7-dimethyl-2-azabicyclo[2.2.2]octan-5-one (**2a**) (13.66 g, 71%) as a light brown solid.

(1*R*,4*S*)-2-(4-Ethoxyphenyl)-7,7-dimethyl-2-azabicyclo[2.2.2]octan-5-one (**2a**) $[\alpha]_D^{12}$ = –85.9 (c = 1.7, $CHCl_3$). ^{1}H NMR (500 MHz, $CDCl_3$) δ 6.90 – 6.76 (m, 2H), 6.69 – 6.50 (m, 2H), 3.97 (q, *J* = 7.0 Hz, 2H), 3.75 (t, *J* = 2.7 Hz, 1H), 3.53 – 3.40 (m, 2H), 2.68 (dd, *J* = 18.9, 2.1 Hz, 1H), 2.62 (p, *J* = 2.8 Hz, 1H), 2.47 (dd, *J* = 18.9, 3.2 Hz, 1H), 1.77 (d, *J* = 3.0 Hz, 2H), 1.38 (t, *J* = 7.0 Hz, 3H), 1.09 (s, 3H), 1.08 (s, 3H); ^{13}C NMR (75 MHz, $CDCl_3$) δ 214.0, 150.5, 143.1, 116.1 (2C), 111.9 (2C), 64.3, 58.2, 47.8, 45.9, 41.2, 38.8, 36.0, 30.1, 28.8, 15.2. HRMS calcd for $C_{17}H_{24}NO_2$ $[M+H]^+$ 274.1807; found 274.1801.

Schmidt Rearrangement in Batch

Schmidt Rearrangement of 2a (Batch mode, Table 1 entry 1).

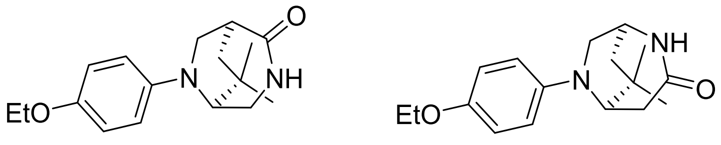

3a and **4a**

To a stirred solution of (1*R*,4*S*)-2-(4-ethoxyphenyl)-7,7-dimethyl-2-azabicyclo[2.2.2]octan-5-one (2.35 g, 8.6 mmol) (**2a**) in concentrated H_2SO_4 (50 mL) at 0 °C was added portionwise sodium azide (650 mg, 10 mmol) (**caution**). After 30 min the mixture was allowed to warm to room temperature and stirring was continued for 2 h. The mixture was cooled to 0 °C and under vigorous stirring was added dropwise (**caution**) 3M NaOH until pH = 9. The mixture was extracted with dichloromethane (3 x 200 mL). The combined organic extracts were dried over anhydrous $MgSO_4$ and concentrated under reduced pressure. Column chromatography (petroleum ether/ethyl acetate) gave (1*S*,5*S*)-6-(4-ethoxyphenyl)-9,9-dimethyl-3,6-diazabicyclo[3.2.2]nonan-2-one (640 mg, 26%) and (1*S*,5R)-6-(4-ethoxyphenyl)-9,9-dimethyl-2,6-diazabicyclo[3.2.2]nonan-3-one (380 mg, 15%).

(1*S*,5*S*)-6-(4-Ethoxyphenyl)-9,9-dimethyl-3,6-diazabicyclo[3.2.2]nonan-2-one (**3a**) ^{1}H NMR (500 MHz, $CDCl_3$) δ 6.88 – 6.82 (m, 2H), 6.68 – 6.61 (m, 2H), 5.48 (s, 1H), 3.97 (q, *J* = 7.0 Hz, 2H), 3.67 (s, 1H), 3.55 (dt, *J* = 10.8, 1.7 Hz, 1H), 3.49 (dt, *J* = 12.6, 2.8 Hz, 1H), 3.44 (ddd, *J* = 12.6, 3.1, 1.8 Hz, 1H), 3.38 (dd, *J* = 10.8, 4.9 Hz, 1H), 2.93 – 2.87 (m, 1H), 1.95 (dt, *J* = 14.3, 1.8 Hz, 1H), 1.65 (dd, *J* = 14.3, 5.9 Hz, 1H), 1.38 (t, *J* = 7.0 Hz, 3H), 1.19 (s, 3H), 1.10 (s, 3H); ^{13}C NMR (75 MHz, $CDCl_3$) δ 177.9, 150.9, 142.9, 116.1 (2C), 112.3 (2C), 64.3, 60.1, 46.5, 43.6, 41.4, 37.2, 35.5, 32.0, 28.6, 15.2. HRMS calcd for $C_{17}H_{25}N_2O_2$ $[M+H]^+$ 289.1916; found 289.1911.

(1*S*,5*R*)-6-(4-Ethoxyphenyl)-9,9-dimethyl-2,6-diazabicyclo[3.2.2]nonan-3-one (**4a**) ^{1}H NMR (500 MHz, $CDCl_3$) δ 7.19 (t, *J* = 9.6 Hz, 1H), 6.91 – 6.82 (m, 2H), 6.68 – 6.51 (m, 2H), 3.97 (q, *J* = 7.0 Hz, 2H), 3.65 – 3.54 (m, 1H), 3.53 – 3.43 (m, 3H), 2.90 – 2.72 (m, 2H), 1.92 (d, *J* = 14.2 Hz, 1H), 1.69 (dd, *J* = 14.3, 4.9 Hz, 1H), 1.37 (t, *J* = 7.0 Hz, 3H), 1.16 (s, 3H), 1.06 (s, 3H); ^{13}C NMR (75 MHz, $CDCl_3$) δ 174.7, 150.8, 143.1, 116.1 (2C), 112.3 (2C), 64.3, 57.4, 51.4, 47.2, 41.8, 36.9, 36.2, 32.0, 29.4, 15.2. HRMS calcd for $C_{17}H_{24}N_2O_2Na$ $[M+Na]^+$ 311.1735; found 311.1721

Beckmann Rearrangement in Batch

Beckmann Rearrangement of 2a (Table 1 entry 10).

To a solution of (1*R*,4*S*)-2-(4-ethoxyphenyl)-7,7-dimethyl-2-azabicyclo[2.2.2]octan-5-one (200 mg, 0.73 mmol) (**2a**) in methanol/CH_2Cl_2 (5:3) (3.5 mL) at room temperature was added portionwise hydroxylamine hydrochloride (61 mg, 0.88 mmol) and the reaction stirred for 3 h. Dichloromethane was added (10 mL) and the organic phase washed with water (2 x 10 mL), brine (5 mL), dried ($MgSO_4$) and concentrated under reduced pressure. ^1H NMR of this crude mixture indicated a 9.1:1 mixure of oxime stereoisomers. The crude mixture was dissolved in THF (5 mL), cooled to 0 ^oC then $SOCl_2$ (59 µL, 0.80 mmol) was added dropwise. The reaction was allowed to warm to rt then stirred for 3 h. Water (5 mL) and EtOAc (5 mL) were added, the phases separated and the aqueous phase extracted with EtOAc (2 x 5 mL). The combined organic phases were washed successively with 10% aq. $NaHCO_3$ (5 mL), brine (5 mL), dried ($MgSO_4$) and concentrated under vacuum. Column chromatography (petroleum ether/ethyl acetate) gave (1*S*,5*R*)-6-(4-ethoxyphenyl)-9,9-dimethyl-2,6-diazabicyclo[3.2.2]nonan-3-one (**4a**) (83 mg, 39%). Analytical data were identical to that obtained from the product of the Schmidt reaction.

Beckmann Rearrangement of 2a (Table 1 entry 11).

To a solution of (1*R*,4*S*)-2-(4-ethoxyphenyl)-7,7-dimethyl-2-azabicyclo[2.2.2]octan-5-one (200 mg, 0.73 mmol) (**2a**) and trimethylamine (122 µL, 0.88 mmol) in methanol/CH_2Cl_2 (5:3) (3.5 mL) at 0 ^oC was added portionwise hydroxylamine hydrochloride (61 mg, 0.88 mmol) and the reaction stirred at 0 ^oC for 3 h. Dichloromethane was added (10 mL) and the organic phase washed with water (2 x 10 mL), brine (5 mL), dried ($MgSO_4$) and concentrated under reduced pressure. ^1H NMR of this crude mixture indicated a 0.32:1 mixture of oxime stereoisomers. The crude mixture was dissolved in THF (5 mL), cooled to 0 ^oC then $SOCl_2$ (59 µL, 0.80 mmol) was added dropwise. The reaction was allowed to warm to rt then stirred for 1.5 h. Water (5 mL) and EtOAc (5 mL) were added, the phases separated and the aqueous phase extracted with EtOAc (2 x 5 mL). The combined organic phases were washed successively with 10% aq. $NaHCO_3$ (5 mL), brine (5 mL), dried ($MgSO_4$) and concentrated under vacuum. Column chromatography (petroleum ether/ethyl acetate) gave (1*S*,5*S*)-6-(4-ethoxyphenyl)-9,9-dimethyl-3,6-diazabicyclo[3.2.2]nonan-2-one (**3a**) (28 mg, 12%) and (1*S*,5*R*)-6-(4-ethoxyphenyl)-9,9-dimethyl-2,6-diazabicyclo[3.2.2]nonan-3-one (**4a**) (40 mg, 19%). Analytical data were identical to that obtained from the products of the Schmidt reaction.

Schmidt Rearrangement in Continuous-Flow

A continuous-flow microreactor was set up consisting of the following components:[4]

Two syringe pumps (Razell-A99), a T-shaped micromixer (ETFE), tubing (PFA, internal diameter: 760 μm) including the microreactor (length: 1.096 m; volume: 500 μL), a water bath (80 °C), a back pressure regulator assembly (BPR, 75 psi) (PEEK) and a collection vessel containing ice and 2M NaOH (1:1).

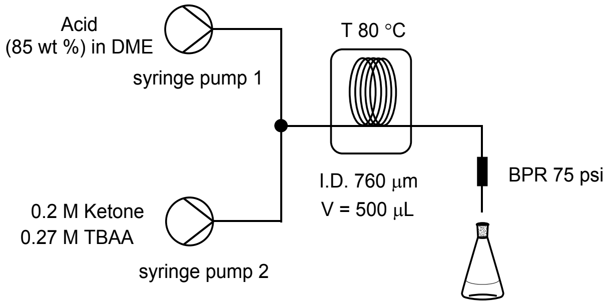

Methanesulfonic acid (MsOH) or concentrated sulfuric acid was diluted to 85 % (w/w) with 1,2-dimethoxyethane (DME) and introduced into syringe 1. Ketone (1.09 g, 4.00 mmol, 0.2 M) and tetrabutylammonium azide (1.54 g, 5.40 mmol, 0.27 M) were dissolved in 1,2-dimethoxyethane and introduced into syringe 2. Operation of the microreactor was carried out behind a blast shield. Syringe pumps were started simultaneously and after steady-state conditions were reached (minimum 3 × t_R) the outflow was collected for the stated time. The material in the collection vessel was brought to pH = 9 with 2M NaOH, then extracted with dichloromethane. The combined organic phases were washed with brine, dried over anhydrous $MgSO_4$ and concentrated under vacuum. Column chromatography (ethyl acetate/petroleum ether) afforded the rearrangement products.

Schmidt Reaction in Continuous-Flow (Table 2 entry 1).

A solution of MsOH (85 %, w/w) in 1,2-dimethoxyethane (flow rate: 3.00 ml.hr^{-1}) and a binary solution of **2a** (0.20 M) and tetrabutylammonium azide (0.27 M) in 1,2-dimethoxyethane (1.50 ml.hr^{-1}) were fed via syringe pump through the microreactor coil (760 µm i.d.; volume: 500 µL) immersed in a water bath at 80 ^{o}C. After steady state conditions were reached (17 min) the outflow was collected for 50 min. The material in the collection vessel was brought to pH = 9 with 2 M NaOH, then extracted with dichloromethane (3 × 30 mL). The combined organic phases were washed with brine (30 mL), dried over anhydrous $MgSO_4$ and concentrated under vacuum. Column chromatography (ethyl acetate/petroleum ether) afforded (1*S*,5*S*)-6-(4-ethoxyphenyl)-9,9-dimethyl-3,6-diazabicyclo[3.2.2]nonan-2-one (**3a**) (7 mg, 8%) and (1*S*,5*R*)-6-(4-ethoxyphenyl)-9,9-dimethyl-2,6-diazabicyclo[3.2.2]nonan-3-one (**4a**) (9 mg, 10%).

(1*S*,5*S*)-6-(4-Ethoxyphenyl)-9,9-dimethyl-3,6-diazabicyclo[3.2.2]nonan-2-one (**3a**) ^{1}H NMR (300 MHz, $CDCl_3$) δ 6.90 – 6.81 (m, 2H), 6.69 – 6.60 (m, 2H), 5.70 (s, 1H), 3.97 (q, *J* = 7.0 Hz, 2H), 3.67 (q, *J* = 2.6 Hz, 1H), 3.59 – 3.33 (m, 4H), 2.95 – 2.86 (m, 1H), 1.95 (dt, *J* = 14.3, 1.9 Hz, 1H), 1.75 – 1.60 (m, 1H), 1.38 (t, *J* = 7.0 Hz, 3H), 1.19 (s, 3H), 1.10 (s, 3H); ^{13}C NMR (75 MHz, $CDCl_3$) δ 177.8, 150.7, 142.8, 116.0 (2C), 112.2 (2C), 64.2, 60.0, 46.4, 43.5, 41.3, 37.1, 35.3, 31.9, 28.5, 15.0. MS (ES^{+}) m/z = 289 ([M+H]$^{+}$, 100).

(1*S*,5*R*)-6-(4-Ethoxyphenyl)-9,9-dimethyl-2,6-diazabicyclo[3.2.2]nonan-3-one (**4a**) ^{1}H NMR (300 MHz, $CDCl_3$) δ 7.11 (d, *J* = 7.3 Hz, 1H), 6.91 – 6.79 (m, 2H), 6.66 – 6.58 (m, 2H), 3.97 (q, *J* = 7.0 Hz, 2H), 3.65 – 3.54 (m, 1H), 3.53 – 3.45 (m, 3H), 2.81 (dd, *J* = 3.7, 1.5 Hz, 2H), 1.92 (d, *J* = 14.3 Hz, 1H), 1.69 (dd, *J* = 14.3, 4.9 Hz, 1H), 1.38 (t, *J* = 7.0 Hz, 3H), 1.16 (s, 3H), 1.07 (s, 3H); ^{13}C NMR (75 MHz, $CDCl_3$) δ 174.7, 150.8, 143.1, 116.1 (2C), 112.3 (2C), 64.3, 57.4, 51.4, 47.2, 41.8, 36.9, 36.2, 32.0, 29.4, 15.2. MS (ES^{+}) m/z = 289 ([M+H]$^{+}$, 100).

Schmidt Reaction in Continuous-Flow (Table 2 entry 2).

A solution of concentrated H_2SO_4 (85 %, w/w) in 1,2-dimethoxyethane (flow rate: 3.00 ml.hr^{-1}) and a binary solution of **2a** (0.20 M) and tetrabutylammonium azide (0.27 M) in 1,2-dimethoxyethane (1.50 ml.hr^{-1}) were fed via syringe pump through the microreactor coil (760 µm i.d.; volume: 500 µL) immersed in a water bath at 80 ^{o}C. After steady state conditions were reached (20 min) the outflow was collected for 1h 45 min. The material in the collection vessel was brought to pH = 9 with 2 M NaOH, then extracted with dichloromethane (3 × 30 mL). The combined organic phases were washed with brine (30 mL), dried ($MgSO_4$) and concentrated under vacuum. Column chromatography (ethyl acetate/petroleum ether) afforded **3a** (25 mg, 16%) and **4a** (27 mg, 17%).

Schmidt Reaction in Continuous-Flow (Table 2 entry 6).

A solution of concentrated H_2SO_4 (85 %, w/w) in 1,2-dimethoxyethane (flow rate: 3.00 ml.hr^{-1}) and a binary solution of **2a** (0.20 M) and tetrabutylammonium azide (0.27 M) in 1,2-dimethoxyethane (0.50 ml.hr^{-1}) were fed via syringe pump through the microreactor coil (760 µm i.d.; volume: 500 µL) immersed in a water bath at 80 ^{o}C. After steady state conditions were reached (17 min) the outflow was collected for 3.5 h. The material in the collection vessel was brought to pH = 9 with 2 M NaOH, then extracted with dichloromethane (3 × 30 mL). The combined organic phases were washed with brine (30 mL), dried ($MgSO_4$) and concentrated under vacuum. Column chromatography (ethyl acetate/petroleum ether) afforded **3a** (40 mg, 40%) and **4a** (39 mg, 39%).

Schmidt Reaction in Continuous-Flow (Table 2 entry 7).

A solution of concentrated H_2SO_4 (85 %, w/w) in 1,2-dimethoxyethane (flow rate: 3.00 ml.hr^{-1}) and a binary solution of **2a** (0.31 M) and tetrabutylammonium azide (0.41 M) in 1,2-dimethoxyethane (0.50 ml.hr^{-1}) were fed via syringe pump through the microreactor coil (760 µm i.d.; volume: 500 µL) immersed in a water bath at 80 ^{o}C. After steady state conditions were reached (17 min) the outflow was collected for 2h 10 min. The material in the collection vessel was brought to pH = 9 with 2 M NaOH, then extracted with dichloromethane (3 × 30 mL). The combined organic phases were washed with brine (30 mL), dried ($MgSO_4$) and concentrated under vacuum. Column chromatography (ethyl acetate/petroleum ether) afforded **3a** (39 mg, 41%) and **4a** (36 mg, 37%).

Photochemical Reactor Setup

A parallel tube flow reactor was constructed as follows. The reactor casing, lamp housing and power supply from a Semi-Micro Photochemical Reactor (RS-55, Photochemical Reactors Ltd., Reading, Berkshire, UK) were used. The cylindrical reflector encasing the lamp was partially removed exposing the UVC bulb (Phillips TUV 4W UVC bulb (λ_{max} = 254 nm)). Perfluoroalkoxy (PFA) tubing (length 15 m; internal diameter 760 µm) was threaded through the reactor casing parallel to and within 50 mm of the bulb such that a 150 mm section of tubing was exposed to the lamp. The tubing was then threaded through the reactor casing antiparallel to the first section of tubing. This procedure was repeated until 16 passes (reactor A, total length exposed to the bulb 2.40 m) or 32 passes (reactor B, total length exposed to the bulb 4.80 m) of the lamp were achieved. The cylindrical reflector was replaced to enclose the lamp and the reactor casing was closed.

The inlet of an HPLC pump (SpectraSYSTEM P4000 Gradient Pump (Thermo Scientific)) was introduced into the vessel containing the oxaziridine solution and the outlet of the pump connected to the PFA tubing. The other end of the PFA tubing was then fed into a collection vessel sealed loosely with laboratory film. The pump and reactor were primed with cyclohexane (5 ml min^{-1}). The diastereomeric mixture of oxaziridines **6** was dissolved in cyclohexane (HPLC) and degassed under stirring for 30 min via a stream of argon bubbled through the solution, then was pumped through the photochemical reactor and collected at the outlet for the specified time. Convective cooling of the reactor (inside a fume cupboard) was sufficient to maintain the temperature in the vicinity of the lamp at 30 ± 5 ^{o}C.

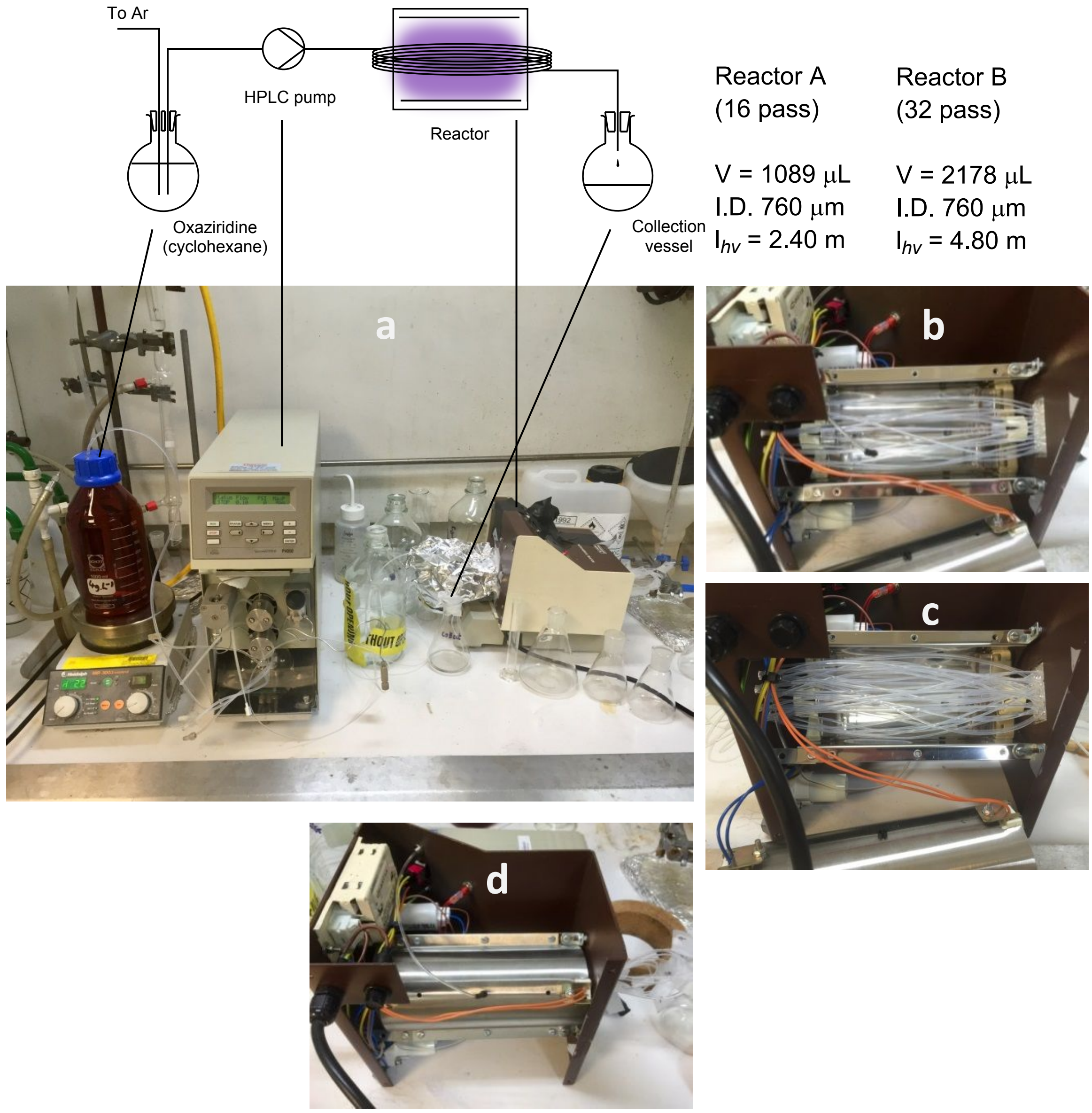

Figure S2. Continuous-flow photochemical microreactor setup. (**a**) Full setup inside a 1.8 m fume cupboard. (**b**) View inside reactor A, 16-pass and (**c**) reactor B, 32-pass with cylindrical metal reflector removed and (**d**) with cylindrical metal reflector replaced.

Preparation of Oxaziridine 6

(1*S*,2*R*,4*R*)-2'-Benzyl-5-(4-ethoxyphenyl)-8,8-dimethyl-5-azaspiro[bicyclo[2.2.2] octane-2,3'-[1,2]oxaziridine] (**6**)

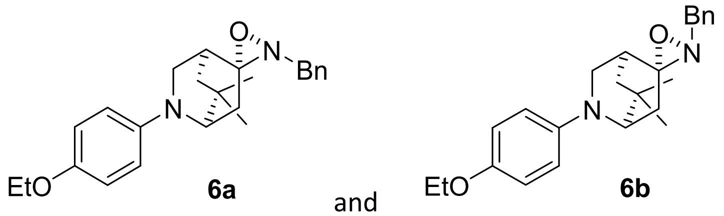

6a and **6b**

To a mixture of (1*R*,4*S*)-2-(4-ethoxyphenyl)-7,7-dimethyl-2-azabicyclo[2.2.2]octan-5-one (**2a**) (5.00 g, 18.29 mmol) and 4 Å molecular sieves (10 g) in anhydrous CH_2Cl_2 (35 mL) was added dropwise benzylamine (2.00 mL, 18.29 mmol) followed by TsOH·H_2O (7 mg, 0.03 mmol) and the mixture allowed to stand for 6 h at room temperature. The reaction was filtered promptly through glass wool to remove the molecular sieves using anhydrous CH_2Cl_2 (25 mL) and under a stream of dry nitrogen gas. The solution was then cooled to −10 °C and a solution of *m*CPBA (≤ 77 wt %, 4.92 g, 21.95 mmol) in anhydrous CH_2Cl_2 (40 mL) was added dropwise under rapid stirring. After the addition, the reaction was maintained at −10 °C for 1 h then allowed to warm to room temperature. The reaction was cooled to 0 °C then quenched by the addition of sat. $Na_2S_2O_3$: sat. $NaHCO_3$ (1:1) (10 mL) followed by water (40 mL). The phases were separated and the aqueous phase extracted with CH_2Cl_2 (2 × 50 mL). The combined organic phases were washed successively with 10% aq. $NaHCO_3$ (50 mL), brine (30 mL), dried ($MgSO_4$) and concentrated under vacuum. Column chromatography (ethyl acetate/cyclohexane) afforded oxaziridine **6** (4.79 g, 69%) as a mixture of diastereoisomers (7.4:1). Analytical samples of the two diastereomers were obtained from early (minor isomer) and late (major isomer) column fractions.

(1*S*,2*R*,2'*s*,4*R*)-2'-Benzyl-5-(4-ethoxyphenyl)-8,8-dimethyl-5-azaspiro[bicyclo[2.2.2] octane-2,3'-[1,2]oxaziridine] (**6a**) *(Major isomer)* ^{1}H NMR (500 MHz, $CDCl_3$) δ 7.43 (d, *J* = 7.3 Hz, 2H), 7.37 (t, *J* = 7.3 Hz, 2H), 7.31 (t, *J* = 7.3 Hz, 1H), 6.85 (d, *J* = 9.1 Hz, 2H), 6.60 (d, *J* = 9.1 Hz, 2H), 3.98 (q, *J* = 6.9 Hz, 2H), 3.90 (app q, *J* = 15.1 Hz, 2H), 3.58 (t, *J* = 2.5 Hz, 1H), 3.51 (dt, *J* = 10.9, 2.5 Hz, 1H), 3.26 (dt, *J* = 9.8, 2.2 Hz, 1H), 2.61 (dd, *J* = 15.5, 2.2 Hz, 1H), 2.26 (dd, *J* = 15.5, 3.5 Hz, 1H), 1.67 (t, *J* = 2.5 Hz, 1H), 1.60 – 1.57 (m, 2H), 1.39 (t, *J* = 6.9 Hz, 3H), 0.98 (s, 3H), 0.89 (s, 3H); ^{13}C NMR (125 MHz, $CDCl_3$) δ 150.0, 143.4, 136.4, 128.8 (2C), 128.7 (2C), 127.8, 116.2 (2C), 111.5 (2C), 86.2, 64.4, 59.4, 55.9, 46.2, 38.5, 37.1, 34.9, 30.1, 29.0, 27.9, 15.2. HRMS calculated for $C_{24}H_{31}N_2O_2$ $[M+H]^+$ 379.2386; found 379.2370.

(1*S*,2*R*,2'*r*,4*R*)-2'-Benzyl-5-(4-ethoxyphenyl)-8,8-dimethyl-5-azaspiro[bicyclo[2.2.2] octane-2,3'-[1,2]oxaziridine] (**6b**) *(Minor isomer)* ^{1}H NMR (500 MHz, $CDCl_3$) δ 7.44 (d, *J* = 7.3 Hz, 2H), 7.38 (t, *J* = 7.3 Hz, 2H), 7.31 (t, *J* = 7.3 Hz, 1H), 6.86 (d, *J* = 9.1 Hz, 2H), 6.61 (d, *J* = 9.1 Hz, 2H), 3.98 (q, *J* = 6.9 Hz, 2H), 3.92 (d, *J* = 14.2 Hz, 1H), 3.71 (d, *J* = 14.2 Hz, 1H), 3.62 (t, *J* = 2.8 Hz, 1H), 3.38 – 3.34 (m, 2H), 2.42 (dq, *J* = 15.5, 3.2 Hz, 2H), 1.83 (dq, *J* = 13.6, 1.9 Hz, 1H), 1.72 (t, *J* = 2.5 Hz, 1H), 1.53 (dt, *J* = 13.6, 2.5 Hz, 1H), 1.39 (t, *J* = 6.9 Hz, 3H), 1.18 (s, 3H), 1.08 (s, 3H); ^{13}C NMR (125 MHz, $CDCl_3$) δ 150.2, 143.3, 136.4, 128.8 (2C), 128.6 (2C), 127.7, 116.1 (2C), 111.9 (2C), 86.0, 64.4, 59.3, 56.5, 47.4, 38.2, 36.4, 35.3, 30.0, 28.7, 27.1, 15.2. HRMS calculated for $C_{24}H_{31}N_2O_2$ $[M+H]^+$ 379.2386; found 379.2363.

Photochemical Rearrangement in Continuous-Flow

Photochemical Rearrangement in Continuous-Flow (Table 3 entry 1).

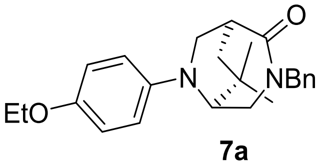

7a and

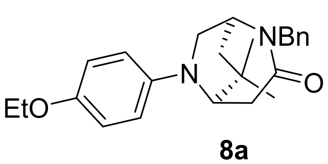

8a

A solution of (1*S*,2*R*,4*R*)-2'-benzyl-5-(4-ethoxyphenyl)-8,8-dimethyl-5-azaspiro[bicyclo[2.2.2] octane-2,3'-[1,2]oxaziridine] **6** (1.20 g, 3.17 mmol) in cyclohexane (305 mL, c = 4.00 g L^{-1}) was pumped through PFA tubing (total length 15.00 m; internal diameter: 760 µm; volume: 6800 µL) including a section irradiated with a 4W UVC bulb (length: 2.40 m, volume: 1089 µL, 760 µm, i.d.) for 17 h via an HPLC pump (flow rate: 0.30 mL min^{-1}, average residence time: 217 s) and collected at the outlet. The solvent was removed under vacuum. Column chromatography (ethyl acetate/cyclohexane) gave (1*S*,5*S*)-3-benzyl-6-(4-ethoxyphenyl)-9,9-dimethyl-3,6-diazabicyclo[3.2.2]nonan-2-one (**7a**) (854 mg, 71%) and (1*S*,5*R*)-2-benzyl-6-(4-ethoxyphenyl)-9,9-dimethyl-2,6-diazabicyclo[3.2.2]nonan-3-one (**8a**) (150 mg, 12%).

(1*S*,5*S*)-3-Benzyl-6-(4-ethoxyphenyl)-9,9-dimethyl-3,6-diazabicyclo[3.2.2]nonan-2-one (**7a**) ^{1}H NMR (500 MHz, $CDCl_3$) δ 7.32-7.24 (m, 5H), 6.86 (d, *J* = 8.8 Hz, 2H), 6.65 (d, *J* = 9.1 Hz, 2H), 4.82 (d, *J* = 14.5 Hz, 1H), 4.26 (d, *J* = 14.5 Hz, 1H), 3.99 (q, *J* = 6.9 Hz, 2H), 3.62 (t, *J* = 3.2 Hz, 1H), 3.58 (d, *J* = 10.7 Hz, 1H), 3.41 (dd, *J* = 5.4, 10.7 Hz, 1H), 3.37 (d, *J* = 3.5 Hz, 2H), 3.17-3.15 (m, 1H), 1.95 (d, *J* = 14.2 Hz, 1H), 1.68 (dd, *J* = 5.7, 14.2 Hz, 1H), 1.39 (t, *J* = 6.9 Hz, 3H), 1.07 (s, 3H), 0.94 (s, 3H); ^{13}C NMR (125 MHz, $CDCl_3$) δ 174.9, 150.7, 142.8, 136.9, 128.6 (2C), 128.3 (2C), 127.5, 115.9 (2C), 112.3 (2C), 64.1, 60.6, 50.6, 49.6, 46.6, 42.1, 37.2, 35.1, 31.6, 27.9, 15.8. HRMS calcd for $C_{24}H_{31}N_2O_2$ $[M+H]^+$ 379.2386; found 379.2364.

(1*S*,5*R*)-2-Benzyl-6-(4-ethoxyphenyl)-9,9-dimethyl-2,6-diazabicyclo[3.2.2]nonan-3-one (**8a**) ^{1}H NMR (500 MHz, $CDCl_3$) δ 7.36-7.30 (m, 5H), 6.85 (d, *J* = 9.1 Hz, 2H), 6.63 (d, *J* = 9.1 Hz, 2H), 4.80 (d, *J* = 14.8 Hz, 1H), 4.53 (d, *J* = 14.8 Hz, 1H), 3.99 (q, *J* = 7.3 Hz, 2H), 3.66-3.64 (m, 1H), 3.51 (t, *J* = 3.8 Hz, 1H), 3.43-3.39 (m, 1H), 3.31 (d, *J* = 11.4 Hz, 1H), 2.93 (dq, *J* = 4.1, 12.9 Hz, 2H), 1.70 (dt, *J* = 2.2, 14.2 Hz, 1H), 1.55 (dd, *J* = 4.7, 14.5 Hz, 1H), 1.39 (t, *J* = 6.9 Hz, 3H), 1.11 (s, 3H), 1.07 (s, 3H); ^{13}C NMR (125 MHz, $CDCl_3$) δ 171.3, 150.9, 142.9, 137.5, 128.7 (2C), 128.4 (2C), 127.6, 115.9 (2C), 112.9 (2C), 64.1, 58.4, 52.6, 51.5, 51.0, 39.8, 37.6, 35.4, 31.5, 28.6, 15.1. HRMS calcd for $C_{24}H_{31}N_2O_2$ $[M+H]^+$ 379.2386; found 379.2368.

Photochemical Rearrangement in Continuous-Flow (Table 3 entry 2).

A solution of oxaziridine **6** (4.04 g, 10.67 mmol) in cyclohexane (1.245 L, c = 3.26 g L^{-1}) was pumped through PFA tubing (total length 15.00 m; internal diameter: 760 µm; volume: 6800 µL) including a section irradiated with a 4W UVC bulb (λ_{max} = 254 nm) (length: 2.40 m, volume: 1089 µL, 760 µm, i.d.) for 26 h via an HPLC pump (flow rate: 0.75 mL min^{-1}, average residence time: 87 s) and collected at the outlet. The solvent was removed under vacuum. Column chromatography (ethyl acetate/cyclohexane) gave (1*S*,5*S*)-3-benzyl-6-(4-ethoxyphenyl)-9,9-dimethyl-3,6-diazabicyclo[3.2.2]nonan-2-one (**7a**) (3.11 g, 82%) and (1*S*,5*R*)-2-benzyl-6-(4-ethoxyphenyl)-9,9-dimethyl-2,6-diazabicyclo[3.2.2]nonan-3-one (**8a**) (414 mg, 11%).

Photochemical Rearrangement in Continuous-Flow (Table 3 entry 3).

A solution of oxaziridine **6** (6.11 g, 16.10 mmol) in cyclohexane (1.875 L, c = 3.26 g L^{-1}) was pumped through PFA tubing (total length 15.00 m; internal diameter: 760 µm; volume: 6800 µL) including a section irradiated with a 4W UVC bulb (λ_{max} = 254 nm) (length: 2.40 m, volume: 1089 µL, 760 µm, i.d.) for 41.5 h via an HPLC pump (flow rate: 0.75 mL min^{-1}, average residence time: 87 s) and collected at the outlet. The solvent was distilled under vacuum and recycled. Column chromatography (ethyl acetate/cyclohexane) gave (1*S*,5*S*)-3-benzyl-6-(4-ethoxyphenyl)-9,9-dimethyl-3,6-diazabicyclo[3.2.2]nonan-2-one (**7a**) (5.11 g, 84%) and (1*S*,5*R*)-2-benzyl-6-(4-ethoxyphenyl)-9,9-dimethyl-2,6-diazabicyclo[3.2.2]nonan-3-one (**8a**) (660 mg, 11%).

Photochemical rearrangement in Continuous-flow (Table 3 entry 4).

A solution of oxaziridine **6** (1.90 g, 5.02 mmol) in cyclohexane (600 mL, c = 3.26 g L^{-1}) was pumped through PFA tubing (total length 15.00 m; internal diameter: 760 µm; volume: 6800 µL) including a section irradiated with a 4W UVC bulb (λ_{max} = 254 nm) (length: 4.80 m, volume: 2178 µL, 760 µm, i.d.) for 8 h via an HPLC pump (flow rate: 1.20 mL min^{-1}, average residence time: 109 s) and collected at the outlet. The solvent was distilled under vacuum and recycled. Column chromatography (ethyl acetate/cyclohexane) gave (1*S*,5*S*)-3-benzyl-6-(4-ethoxyphenyl)-9,9-dimethyl-3,6-diazabicyclo[3.2.2]nonan-2-one (**7a**) (1.61 g, 85%) and (1*S*,5*R*)-2-benzyl-6-(4-ethoxyphenyl)-9,9-dimethyl-2,6-diazabicyclo[3.2.2]nonan-3-one (**8a**) (183 mg, 10%).

Reduction and Deprotection to Homopiperazines 1a-c

(1*R*,5*S*)-3-Benzyl-6-(4-ethoxyphenyl)-9,9-dimethyl-3,6-diazabicyclo[3.2.2]nonane (1b)

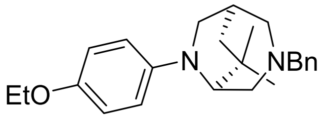

To a solution of (1*S*,5*S*)-3-benzyl-6-(4-ethoxyphenyl)-9,9-dimethyl-3,6-diazabicyclo[3.2.2]nonan-2-one (5.43 g, 14.35 mmol) in anhydrous THF (50 mL) at 0 °C was added dropwise $LiAlH_4$ solution (1.0 M in THF, 28.72 mL, 28.72 mmol) and the reaction heated at reflux for 30 min. After allowing to cool the reaction was quenched by the addition of water (1.4 mL) followed by 2 M NaOH (2.5 mL). Dried $MgSO_4$ was added, the reaction was filtered, then concentrated under vacuum to give (1*R*,5*S*)-3-benzyl-6-(4-ethoxyphenyl)-9,9-dimethyl-3,6-diazabicyclo[3.2.2]nonane (**1b**) (5.08 g, 97%) which was used without purification.

^{1}H NMR (500 MHz, $CDCl_3$) δ 7.38-7.33 (m, 4H), 7.30-7.27 (m, 1H), 6.88 (d, *J* = 9.1 Hz, 2H), 6.56 (d, *J* = 9.1 Hz, 2H), 4.01 (q, *J* = 6.9 Hz, 2H), 3.58 (d, *J* = 12.9 Hz, 1H), 3.50 (d, *J* = 12.9 Hz, 1H), 3.31 (d, *J* = 5.0 Hz, 1H), 3.28 (dd, *J* = 10.1, 2.5 Hz, 1H), 3.16-3.10 (m, 2H), 3.05-3.01 (m, 1H), 2.38 (d, *J* = 11.4 Hz, 1H), 2.33-2.31 (m, 1H), 2.23 (d, *J* = 11.7 Hz, 1H), 2.00 (d, *J* = 12.9 Hz, 1H), 1.41 (t, *J* = 6.9 Hz, 3H), 1.37-1.33 (m, 1H), 1.22 (s, 3H), 0.86 (s, 3H); ^{13}C NMR (125 MHz, $CDCl_3$) δ 149.5, 144.5, 139.6, 129.0 (2C), 128.2 (2C), 126.9, 116.0 (2C), 110.8 (2C), 64.3, 62.6, 60.8, 59.9, 58.0, 50.1, 36.7 (2C), 32.7, 32.4, 28.7, 15.2. HRMS calcd for $C_{24}H_{33}N_2O$ $[M+H]^+$ 365.2593; found 365.2590.

(1*R*,5*S*)-6-(4-Ethoxyphenyl)-9,9-dimethyl-3,6-diazabicyclo[3.2.2]nonane (1a)

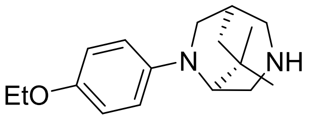

To a solution of (1*R*,5*S*)-3-benzyl-6-(4-ethoxyphenyl)-9,9-dimethyl-3,6-diazabicyclo[3.2.2]nonane (4.39 g, 12.04 mmol) in 2,2,2-trifluoroethanol (50 mL) was added $Pd(OH)_2$ (10 % w/w, 439 mg, 10 wt %). The solution was degassed with nitrogen then heated at reflux under an atmosphere of hydrogen for 20 h. The reaction was allowed to cool then filtered through Celite (ethanol). The filtrate was concentrated under vacuum. Column chromatography (ethyl acetate/cyclohexane) gave (1*R*,5*S*)-6-(4-ethoxyphenyl)-9,9-dimethyl-3,6-diazabicyclo[3.2.2]nonane (**1a**) (2.88 g, 87%).

^{1}H NMR (500 MHz, $CDCl_3$) δ 6.85 (d, *J* = 9.1 Hz, 2H), 6.56 (d, *J* = 9.1 Hz, 2H), 3.98 (q, *J* = 6.9 Hz, 2H), 3.32-3.31 (m, 1H), 3.29 (dd, *J* = 10.1, 2.1 Hz, 1H), 3.24 (app dd, *J* = 13.6, 3.8 Hz, 1H), 3.14 (dt, *J* = 10.1, 2.8 Hz, 1H), 3.10-3.06 (m, 1H), 2.88 (dd, *J* = 13.2, 2.1 Hz, 1H), 2.84 (d, *J* = 13.6, 1H), 2.32-2.20 (m, 1H), 1.77 (br s, 1H) 1.71 (d, *J* = 13.6 Hz, 1H), 1.49-1.45 (m, 1H), 1.38 (t, *J* = 6.9 Hz, 3H), 1.15 (s,

3H), 0.89 (s, 3H); ^{13}C NMR (125 MHz, $CDCl_3$) δ 149.7, 144.4, 116.0 (2C), 110.8 (2C), 64.3, 62.9, 53.8, 49.4, 49.1, 36.9, 36.5, 34.0, 32.5, 29.2, 15.1. HRMS calcd for $C_{17}H_{27}N_2O$ $[M+H]^+$ 275.2123; found 275.2119.

***tert*-Butyl (1*R*,5*R*)-3-benzyl-9,9-dimethyl-3,6-diazabicyclo[3.2.2]nonane-6-carboxylate (1c)**

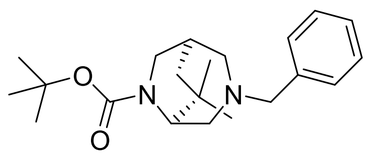

To a solution of (1*R*,5*S*)-3-benzyl-6-(4-ethoxyphenyl)-9,9-dimethyl-3,6-diazabicyclo[3.2.2]nonane (2.44 g, 6.70 mmol) in acetonitrile (30 mL) and water (10 mL) at 0 °C was added portionwise ammonium cerium(IV) nitrate (16.58 g, 30.24 mmol). The reaction was maintained at 0 °C for 3 h then allowed to room temperature and stirred until complete conversion (20 h). The acetonitrile was removed in vacuo then the reaction cooled to 0 °C and quenched by the dropwise addition of an aqueous mixture of sat. $Na_2S_2O_3$: sat. $NaHCO_3$ (1:1) (30 mL) followed by 2 M NaOH (10 mL). Dichloromethane (40 mL) was added then di-*tert*-butyldicarbonate (2.98 g, 13.65 mmol) and 4-(dimethylamino)pyridine (16 mg, 0.13 mmol) was added and the biphasic mixture stirred at rt for 24 h. The reaction was filtered through celite (CH_2Cl_2). The phases were separated and the aqueous phase basified to pH >9 with 2M NaOH then extracted with dichloromethane (3 × 50 mL). The combined organic phases were washed with brine (30 mL), dried ($MgSO_4$) and concentrated under vacuum. Column chromatography (ethyl acetate/cyclohexane) gave *tert*-butyl (1*R*,5*R*)-3-benzyl-9,9-dimethyl-3,6-diazabicyclo[3.2.2]nonane-6-carboxylate as a yellow oil (1.96 g, 85%).

^{1}H NMR (300 MHz, $CDCl_3$) (observed as a mixture of rotamers) δ 7.39 – 7.19 (m, 5H), 3.77 – 3.55 (m, 1H), 3.55 – 3.33 (m, 3H), 3.21 – 3.12 (m, 1H), 3.11 – 2.90 (m, 2H), 2.31 – 2.21 (m, 1H), 2.21 – 2.02 (m, 2H), 1.87 (d, *J* = 13.1 Hz, 1H), 1.54 – 1.41 (m, 9H), 1.25 (ddd, *J* = 13.1, 9.3, 6.3 Hz, 1H), 1.14 (d, *J* = 3.9 Hz, 3H), 0.93 (s, 3H); ^{13}C NMR (75 MHz, d_6-DMSO) (observed as a mixture of rotamers) δ 154.9 (154.4), 138.7, 128.7 (2C), 128.1 (2C), 126.9, 78.2, 61.5, 59.5 (58.8), 57.3, 47.8, 47.3, 36.5, 34.5, 31.7 (31.5), 30.9 (30.7), 28.2 (3C), 27.8. HRMS calcd for $C_{21}H_{33}N_2O_2$ $[M+H]^+$ 345.2542; found 345.2528.

Determination of Enantiomeric Excess of 1a

(1*S*,5*S*)-6-(4-Ethoxyphenyl)-*N*-(2-hydroxyethyl)-9,9-dimethyl-3,6-diazabicyclo[3.2.2]nonane-3-carboxamide (**S-1**)

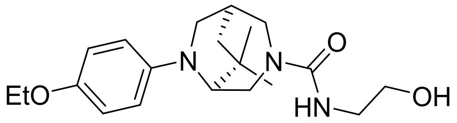

S-1

(1*R*,5*S*)-6-(4-Ethoxyphenyl)-9,9-dimethyl-3,6-diazabicyclo[3.2.2]nonane (**1a**) was derivatized as follows. To a stirred solution of (1*R*,5*S*)-6-(4-ethoxyphenyl)-9,9-dimethyl-3,6-diazabicyclo[3.2.2]nonane (**1a**) (100 mg, 0.36 mmol) in CH_2Cl_2 (5 mL) at 0 °C was added triethylamine (254 µL, 1.82 mmol) and triphosgene (152 mg, 0.51 mmol). The reaction was stirred at 0 °C for 15 min then 2-aminoethanol (500 µL, 8.19 mmol) was added dropwise. The mixture was stirred at room temperature for 72 h then diluted with CH_2Cl_2 and washed with 5% aq NH_4Cl (2 × 10 mL), sat. aq. $NaHCO_3$ (2 × 10 mL) then brine (10 mL). The organic layer was dried ($MgSO_4$) and concentrated under vacuum. Column chromatography (ethyl acetate/methanol) gave (1*S*,5*S*)-6-(4-ethoxyphenyl)-*N*-(2-hydroxyethyl)-9,9-dimethyl-3,6-diazabicyclo[3.2.2]nonane-3-carboxamide (**S-1**) (117 mg, 90%).

^1H NMR (500 MHz, $CDCl_3$) δ 6.84 (d, *J* = 9.1 Hz, 2H), 6.55 (d, *J* = 9.1 Hz, 2H), 5.06 (t, *J* = 5.4 Hz, 1H), 4.23 (dd, *J* = 13.4 and 8.0 Hz, 1H), 4.09 (dd, *J* = 12.6, 3.3 Hz, 1H), 3.96 (q, *J* = 7.0 Hz, 2H), 3.72 – 3.66 (m, 2H), 3.51 (br s, 1H), 3.44 – 3.35 (m, 3H), 3.28 (dd, *J* = 10.2, 2.3 Hz, 1H), 3.18 – 3.14 (m, 2H), 2.96 (d, *J* = 13.6 Hz, 1H), 2.48 (t, *J* = 6.8 Hz, 1H), 1.59 (d, *J* = 13.8 Hz, 1H), 1.43 (dd, *J* = 13.7, 6.5 Hz, 1H), 1.37 (t, *J* = 7.0 Hz, 3H), 1.04 (s, 3H), 0.88 (s, 3H); ^{13}C NMR (125 MHz, $CDCl_3$) δ 159.7, 150.2, 144.0, 116.1 (2C), 111.2 (2C), 64.3, 63.7, 61.3, 50.5, 49.3, 48.5, 44.0, 36.3, 35.8, 32.4, 32.2, 29.2, 15.1. HRMS calcd for $C_{20}H_{32}N_3O_3$ $[M+H]^+$ 362.2438; found 362.2439.

3µL standard injections (with needle wash) of the sample were made onto a Phenomenex Lux Cellulose-2 column (3µm, 250 x 10mm, Phenomenex, Torrance, CA, USA). Solvents were degassed on a 1200 series degasser (Agilent, Santa Clara, USA). Chromatographic separation at room temperature was carried out using a 1200 Series HPLC (Agilent, Santa Clara, USA) over a 15 minute isocratic elution at 90% Methanol / 10% Isopropyl Alcohol at a flow rate of 0.75mL/min. UV-Vis spectra were acquired at 254nm on a 1200 Series diode array detector (Agilent, Santa Clara, USA). Raw data was processed using Agilent Chemstation software (version B.03.01).

Analysis of Enantiomer I (from L-proline) and Enantiomer II (from D-proline) indicated enantiomeric excess of 99.2% and 95.6% respectively.

Solution	t_R = 6.0mins	t_R = 8.9mins	ee
Racemate	51.4%	48.6%	N/A
Enantiomer II *(from D-proline)*	97.8%	2.2%	95.6%
Enantiomer I *(from L-proline)*	0.4%	99.6%	99.2%

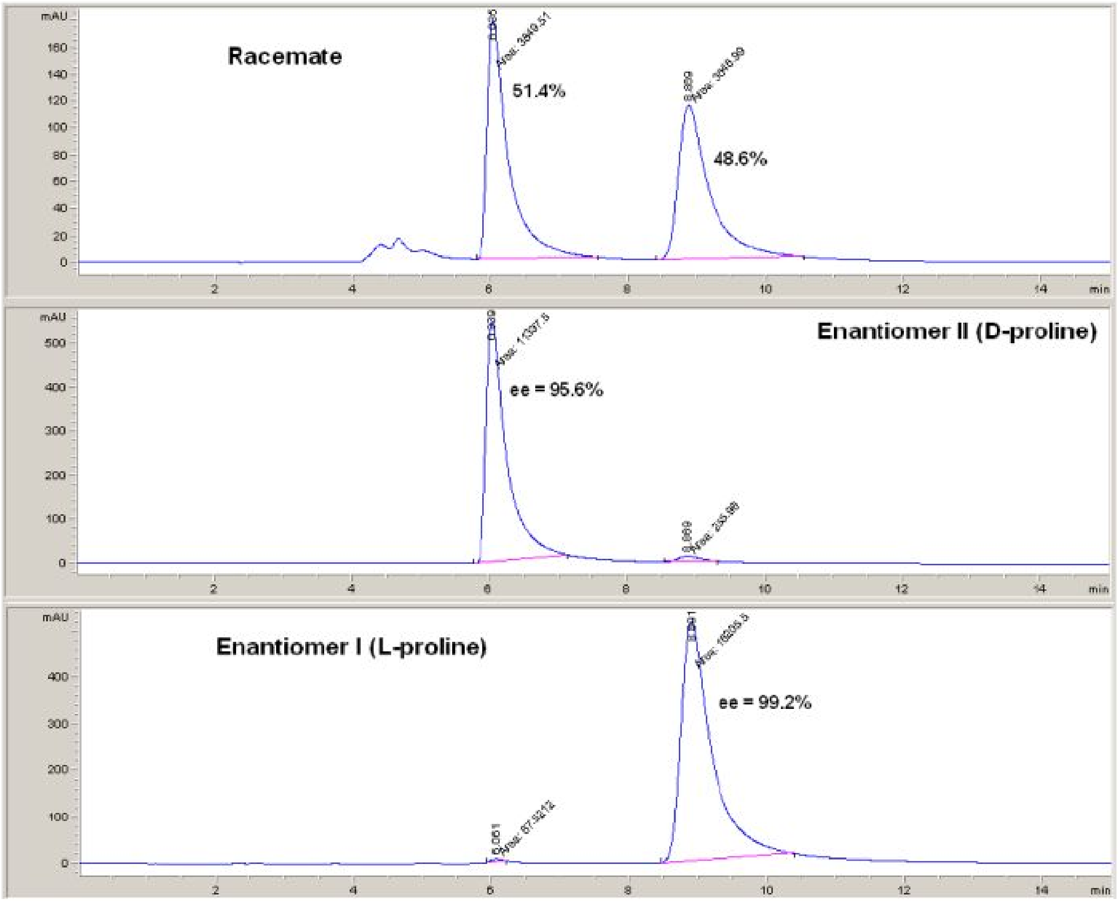

References

(1) Oliveros, E.; Riviere, M.; Malrieu, J. P.; Teichteil, C. Theoretical Exploration of the Photochemical Rearrangement of Oxaziridines. *J. Am. Chem. Soc.* **1979**, *101* (2), 318–322.

(2) Taniguchi, M.; Lindsey, J. S. Database of Absorption and Fluorescence Spectra of >300 Common Compounds for Use in PhotochemCAD. *Photochem. Photobiol.* **2018**, *94* (2), 290–327.

(3) Sundén, H.; Ibrahem, I.; Eriksson, L.; Córdova, A. Direct Catalytic Enantioselective Aza-Diels-Alder Reactions. *Angew. Chem. Int. Ed.* **2005**, *44* (31), 4877–4880.

(4) Chen, Y.; Liu, B.; Liu, X.; Yang, Y.; Ling, Y.; Jia, Y. Schmidt Reaction of Ketones in DME Solution in a Continuous-Flow Microreactor. *Org. Process Res. Dev.* **2014**, *18* (11), 1589–1592.
